# Substantial gradient mitigation in simulated large‐scale bioreactors by optimally placed multiple feed points

**DOI:** 10.1002/bit.28232

**Published:** 2022-09-26

**Authors:** Pauli Losoi, Jukka Konttinen, Ville Santala

**Affiliations:** ^1^ Faculty of Engineering and Natural Sciences Tampere University Tampere Finland

**Keywords:** bioreactor, compartment model, feed point, industrial biotechnology, mixing time, scale‐up

## Abstract

The performance of large‐scale stirred tank and bubble column bioreactors is often hindered by insufficient macromixing of feeds, leading to heterogeneities in pH, substrate, and oxygen, which complicates process scale‐up. Appropriate feed placement or the use of multiple feed points could improve mixing. Here, theoretically optimal placement of feed points was derived using one‐dimensional diffusion equations. The utility of optimal multipoint feeds was evaluated with mixing, pH control, and bioreaction simulations using three‐dimensional compartment models of four industrially relevant bioreactors with working volumes ranging from 8 to 237 m^3^. Dividing the vessel axially in equal‐sized compartments and locating a feed point or multiple feed points symmetrically in each compartment reduced the mixing time substantially by more than a minute and mitigated gradients of pH, substrate, and oxygen. Performance of the large‐scale bioreactors was consequently restored to ideal, homogeneous reactor performance: oxygen consumption and biomass yield were recovered and the phenotypical heterogeneity of the biomass population was diminished.

## Graphical abstract

In this study, Losoi, Konttinen, and Santala derived optimal feed point placements for large‐scale bioreactors using one‐dimensional diffusion equations. The placements were evaluated with three‐dimensional compartment model simulations of both stirred tank (8 and 24 m^3^) and bubble column (40 and 237 m^3^) bioreactors. The use of multiple optimal feed points instead of the common top feed improved both mixing and bioreaction substantially.

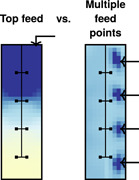

## 1 INTRODUCTION

The transfer of a bioprocess from laboratory toward industrial scale would be relatively simple, if the large‐scale reactors behaved exactly as the laboratory‐scale reactors. However, the correspondence is far from perfect, which manifests itself as heterogeneity with respect to feed(s) (Bylund et al., 1998; Langheinrich & Nienow, 1999; Larsson et al., 1996; Xu et al., 1999) and insufficient oxygen transfer at the large scale (Oosterhuis & Kossen, 1984; Xu et al., 1999). For example, 10%–20% lower *Escherichia coli* biomass yields have been reported as a consequence in large‐scale aerobic fed‐batch processes (Bylund et al., 1998; Xu et al., 1999). Scale‐up could be facilitated by improving the various modeling approaches to better estimate the large‐scale behavior and the scale‐up losses beforehand. As far as hydrodynamics are concerned, such predictive modeling is nowadays quite feasible with computational fluid dynamics (CFD) simulations of model fluids (water, air). However, the behavior of biomass is insufficiently known to be reliably predicted at a heterogeneous large scale. Another option would be to homogenize the large‐scale reactor more efficiently to make it resemble the better‐performing, more predictable small‐scale reactors. This approach would have the advantage that both the bioreaction kinetics determined at the laboratory scale and the ideal homogeneous reactor model would retain their validity also at the larger scale.

Experiments suggest that feed point placement has a marked effect on mixing: feed at middle height mixes most rapidly and feed at the top most slowly (Alves et al., 1997; Cronin et al., 1994; Vrábel et al., 1999). Correspondingly the substrate gradients are milder when the feed is brought to a bottom impeller instead of the stagnant top (Bylund et al., 1998; Larsson et al., 1996), a conventional choice. The use of multiple feed points has been suggested as well (Bylund et al., 1998; Cronin et al., 1994; Enfors et al., 2001; Larsson et al., 1996), and mixing indeed improves by appropriate placement of two feed points (Fu et al., 2005). The enhanced mixing has important consequences for bioreaction: yeast yields have been improved by placing the feed close to an impeller (Dunlop & Ye, 1990) or by using multiple feed points (Hansford & Humphrey, 1966). Numerical simulations have also shown similar improvements in both mixing and bioreaction upon relocating the feed from the top to vicinity of an impeller (Haringa et al., 2018; Morchain et al., 2014).

Upon considering multipoint feeds, the question is how they should be placed in a bioreactor for optimum performance. Literature suggests placement close to impellers or to otherwise well‐mixed zones (Bylund et al., 1998; Cronin et al., 1994; Enfors et al., 2001; Larsson et al., 1996), but a general theoretically optimal placement is not defined. Earlier work on the subject relied on a brute‐force search with case‐specific compartment models (Fu et al., 2005), which suffers from exponential combinatorics as the number of feed points is increased. The aims of this work were to derive general and theoretically optimal feed point placements and to evaluate their relevance in mitigating the gradients found in large‐scale bioreactors. The simple and general one‐dimensional (1D) diffusion equation that has successfully described axial mixing in various high aspect ratio bioreactors (Kasat & Pandit, 2004; Kawase, 1989; Machon & Jahoda, 2000) was studied to deduce the optimal placements, and the effect of multiple optimally placed feed points on mixing and bioreaction was simulated in industrial‐scale stirred tank and bubble column bioreactors with three‐diemsnisonal (3D) compartment models.

## 2 COMPUTATIONAL METHODS

### 2.1 Reactor models

**Figure 1:**
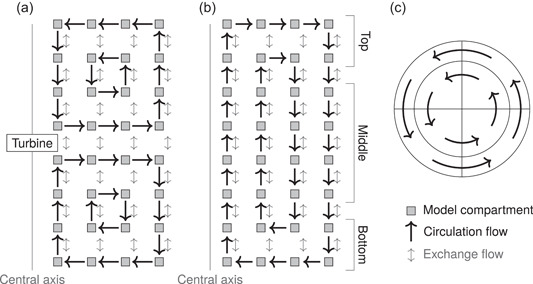
Compartment models. (a) Two square networks of nested loops (two in figure) composed the radial flow pattern induced by Rushton turbines in reactors R4 and R1. (b) The flow pattern in bubble columns was composed of a rectangular network of nested loops (two in figure). Axially adjacent compartments were connected by exchange flows in both reactor types. Conceptually the bubble column was composed of top, middle, and bottom parts such that the middle part contained only vertical circulation flows. (c) Tangentially adjacent compartments in stirred tanks were unidirectionally connected by circulation flows. In bubble columns, exchange flows were used instead of circulation flows.

Four experimentally studied bioreactors were chosen from the literature for the numerical experiments. The reactors are listed in Table 1, and they cover industrially relevant stirred tanks and bubble columns with working volumes from 8 to $237\;m^{3}$ in different geometries and configurations with gas flow rates ranging from 0.085 to 0.98 vvm (vvm: volume flow rate of gas per volume of liquid per minute). Each reactor was simulated with a compartment model based on previously published modeling approaches. Figure 1 illustrates the used compartment models. Model details are given in Appendix A.

**Table 1:** Modeled reactors

Reactor	T	V	H∕T	$\epsilon_{\;\text{G}\;}$	$u_{\;\text{G}\;}$	$N_{\;\text{i}\;}$	D∕T	n	
	m	$m^{3}$	−	%	$\text{cm}\;s^{-1}$	−	−	rpm	References
Stirred tanks									
R4	2.09	23.8	3.33	5.90	0.923	4	1/3	115	Cui et al. (1996)
R1	2.00	8.17	1.30	0	0	1	2/9	60	Langheinrich et al. (1998)
Bubble columns									
B13	1.60	40.2	12.5	23.3	25.0				Schügerl (1993)
B6	3.70	237	5.95	17.0	6.50				Zahradník et al. (2001)

*Note*: Symbols: T, tank diameter; V, working volume; H, working height; $\epsilon_{\;\text{G}\;}$, gas‐holdup; $u_{\;\text{G}\;}$, superficial gas velocity at reactor's half‐height; $N_{\;\text{i}\;}$, number of impellers; D, impeller diameter; n, stirrer speed.

### 2.2 Simulations

Three different situations were simulated in the large‐scale bioreactors (Table 1) with the compartment models to evaluate the multiple feed point placements: (1) pulse addition of a tracer, (2) pulse of a pH‐controlling alkaline agent, and (3) bioreaction with a Monod‐type substrate consumption rate. The substrate concentration fields determined in case 3 were furthermore used to separately estimate the effects on dissolved oxygen concentration, substrate consumption, biomass yield, and adaptation of biomass‐specific growth and substrate uptake rates. The model substrate was considered to be glucose. The bioreaction was simulated as a pseudosteady‐state snap‐shot (Hristov et al., 2001) of a fed‐batch culture with the substrate consumption rate ($g\;L^{-1}\;h^{-1}$)

(1) $$r_{S}=q_{S}\frac{S}{S+K_{S}}X,$$

where $q_{S}$ is the biomass‐specific maximal substrate‐consumption rate ($g\;g^{-1}\;h^{-1}$), S the substrate's concentration ($g\;L^{-1}$), $K_{S}$ the affinity constant ($g\;L^{-1}$), and X the biomass concentration ($10\;g\;L^{-1}$). The substrate feed rate was 4 $g\;L^{-1}\;h^{-1}$ in the bioreaction simulations and it was modeled as a volumetric source in the compartment(s) closest to the feed coordinate(s). Table 2 lists the values for all kinetic parameters used in the simulations.

**Table 2:** Kinetic parameters

Parameter	Value	Unit	Source
X	10	$g_{X}\;L^{-1}$	10; Morchain et al. (2014)
$q_{S}$	1	$g_{S}\;g_{X}^{-1}\;h^{-1}$	1.25; Xu et al. (1999)
			0.6356; Anane et al. (2017)
$q_{\;\text{d}\;}$	0.025	$g_{X}\;g_{X}^{-1}\;h^{-1}$	0.04; Xu et al. (1999)
			0.0129; Anane et al. (2017)
$K_{S}$	0.025	$g_{S}\;L^{-1}$	0.05; Xu et al. (1999)
			0.0370; Anane et al. (2017)
$K_{O}$	0.1	${\text{mg}}_{O}\;L^{-1}$	0.1; Morchain et al. (2013)
$Y_{XS}$	0.5	$g_{X}\;g_{S}^{-1}$	0.51; Xu et al. (1999)
			0.49; Xu et al. (1999)
$Y_{OS}$	1.067	$g_{O}\;g_{S}^{-1}$	Stoichiometry
$T_{X}$	2.5	h	$1.25∕\left(Y_{XS}q_{S}\right)$; Morchain and Fonade (2009); Morchain et al. (2013)
$T_{S}$	0.025	h	$0.01T_{X}$; Morchain et al. (2014)
Δμ	0.05	$g_{X}\;g_{X}^{-1}\;h^{-1}$	$Y_{XS}q_{S}∕10$
Δq	0.10	$g_{S}\;g_{X}^{-1}\;h^{-1}$	$q_{S}∕10$
$k_{\;\text{L}\;}a$	180	$h^{-1}$	180; Xu et al. (1999)
$O_{\;\text{e}\;}$	12.69	${\text{mg}}_{O}\;L^{-1}$	Estimated

*Note*: Symbols: X, biomass concentration; $q_{S}$, biomass‐specific maximal substrate uptake rate; $q_{\;\text{d}}$, biomass‐specific decay rate; $K_{S}$, substrate affinity constant; $K_{O}$, oxygen affinity constant; $Y_{XS}$, maximal biomass yield on substrate; $Y_{OS}$, oxygen demand of substrate; $T_{X}$, adaptation time‐scale of growth; $T_{S}$, adaptation time‐scale of substrate uptake; Δμ, discretization of specific growth rate; Δq, discretization of specific substrate uptake rate; $k_{\;\text{L}\;}a$, oxygen transfer coefficient; $O_{\;\text{e}}$, oxygen solubility.

#### 2.2.1 Mixing time

Mixing times were simulated with various combinations of feed points by the pulse addition of a tracer. Tracer injection was always initialized at the compartment(s) closest to the feed coordinate(s). To circumvent the effects of probe placement on mixing time (Cui et al., 1996), the simulated mixing times were defined in a global sense by monitoring the standard deviation of the dimensionless tracer concentration ($u=C∕\left(V^{-1}\int_{V}C\;\text{d}\;V\right)$) in the whole modeled volume V:

(2) $$\sigma \left(t\right)=\sqrt{\int_{V}{\left(1-u\left(t\right)\right)}^{2}\;\text{d}\;V}.$$

A 95% mixing time $t_{95}$ is commonly determined in point‐probe experiments (e.g., Cui et al., 1996; Vrábel et al., 1999), and here the threshold for simulated mixing time was similarly set to $\sigma \left(t_{95}\right)=5\%$ despite the inherent differences in local and volume‐based mixing time quantification. Unless explicitly stated otherwise, the presented (Equation 2) volume‐based definition of mixing time is used.

A complementary measure of macromixing performance was obtained by calculating an inhomogeneity number (Mayr et al., 1992).

(3) $$N_{\;\text{I}\;}=\sigma \left(t_{95}∕2\right),$$

which is the median of the monotonously decreasing standard deviation in the mixing time interval $t\in \left[0,t_{95}\right]$. It is possible that two feed arrangements have practically identical mixing times $t_{95}$, but in such a scenario, the lower inhomogeneity number $N_{\;\text{I}\;}$ implies more homogeneous reactor contents during the time interval and thus better mixing performance. Compared to the definition used by Mayr et al. (1992), standard deviation was used here instead of mean absolute deviation, the time interval's end was set to the mixing time threshold, and the median of deviation was calculated instead of the mean. Unlike the mean, the median is robust to the inevitable differences in initial conditions (t=0), which are due to unequal feed compartment sizes within and across the modeled reactors.

#### 2.2.2 pH‐control

The effect of feed points on pH‐control (situation 2) was simulated with a 100 $\text{mmol}\;L^{-1}$ carbonate buffer solution ($\;{\text{pK}}_{\text{a}\;}=6.35$) initially at a pH of 4.8. A pulse of carbonate was then added to raise the overall pH to 6, and the evolution of pH was monitored for 10 s. The local pHs were calculated from the local carbonate concentrations $\left[{\;\text{CO}\;}_{3}^{2-}\right]$ as

(4) $$\text{pH}\;={\text{pK}}_{\text{a}\;}+\log_{10}\frac{\left[\;\text{CO}{\;}_{3}^{2-}\right]}{\left[\;\text{HCO}{\;}_{3}^{-}\right]}.$$

Owing to the pH's nonlinear definition, its volumetric mean was time‐dependent even though the carbonate concentration's mean was not.

#### 2.2.3 Dissolved oxygen

The consumption of oxygen along with substrate was modeled only in R4 (experimentally determined oxygen transfer data not available for others) by setting the dissolved oxygen consumption rate ($\text{mg}\;L^{-1}\;h^{-1}$) to

(5) $$r_{O}=Y_{\mathrm{OS}}\frac{O}{O+K_{O}}r_{S},$$

where $Y_{\mathrm{OS}}$ is the oxygen mass required for the aerobic consumption of a substrate (glucose) mass ($g\;g^{-1}$), O the dissolved oxygen concentration ($\text{mg}\;L^{-1}$), and $K_{O}$ the affinity constant for oxygen ($\text{mg}\;L^{-1}$). Equation (5) implies that the aerobically respired proportion of substrate is $O∕\left(O+K_{O}\right)$ of the total consumption. Oxygen was provided by oxygen transfer at a volumetric rate ($\text{mg}\;L^{-1}\;h^{-1}$) of

(6) $$k_{\;\text{L}\;}a\left(O_{\;\text{e}\;}-O\right),$$

where the oxygen transfer coefficient was $k_{\;\text{L}\;}a=180\;h^{-1}$ as measured in the R4 reactor (Xu et al., 1999) at the same operating conditions as simulated here (115 rpm stirrer speed, $35^{\circ }C$ temperature, 1.5 bar head‐space pressure). To focus solely on the effect of feed point placement, oxygen was not modeled in the gas phase, but a homogeneous oxygen solubility $O_{\;\text{e}\;}$ ($\text{mg}\;L^{-1}$) was assumed throughout the reactor (equal partial pressure of oxygen in the whole volume). The effect of hydrostatic and head‐space pressures on the overall oxygen solubility was taken into account, however, and the spatially homogeneous equilibrium concentration of oxygen ($O_{\;\text{e}\;}$) was taken at the partial pressure of oxygen (21% of air) at the reactor's middle height (water density 994 $\text{kg}\;m^{-3}$). At the $35^{\circ }C$ temperature, the dimensionless Henry's constant (concentration in liquid/concentration in gas) for oxygen was 0.0266 (Sander, 2015).

#### 2.2.4 Time‐scale of substrate consumption

The local time‐scales $\tau_{S}$ ($s^{-1}$) of substrate consumption were calculated by linearizing the substrate uptake rate (Equation 1) with respect to the substrate concentration ($r_{S}=S∕\tau_{S}$) and taking the inverse of the first‐order rate‐pseudoconstant $1∕\tau_{S}$:

(7) $$\tau_{S}=\frac{S+K_{S}}{q_{S}X}.$$

#### 2.2.5 Biomass yield

An instantaneous nonconstant biomass yield $y_{XS}$ ($g\;g^{-1}$) was calculated by assuming a maximal yield $Y_{XS}$ of biomass on the substrate and a first‐order decay of biomass at a specific rate ($h^{-1}$) of $q_{\;\text{d}\;}$ (similar to Anane et al., 2017; Xu et al., 1999):

(8) $$y_{XS}=\frac{Y_{XS}r_{S}-q_{\;\text{d}}X}{r_{S}}=Y_{XS}-\frac{q_{\text{d}\;}}{q_{S}}\left(1+\frac{K_{S}}{S}\right).$$

#### 2.2.6 Adaptation of biomass‐specific rates

A population balance was used to simulate the adaptation of biomass‐specific growth and substrate uptake rates similarly to Morchain et al. (2013, 2014). In short, the 10 $g\;L^{-1}$ total biomass was conceptually divided into 10 classes, each with their own specific growth rate ($h^{-1}$) $\mu_{i}=Y_{XS}q_{S}\left(2i-1\right)∕20$, where i∈1,2,…,10. Each of the classes then represented the amount of biomass growing at the specific growth rate characteristic to the class. For example, if each of the 10 classes contained 1 $g\;L^{-1}$ biomass, 10% of the whole population would grow at a rate of 0.025 $h^{-1}$, 10% at 0.075 $h^{-1}$, and so on, and the population's averaged specific growth rate would be 0.25 $h^{-1}$. The transfer rate ($g\;L^{-1}\;h^{-1}$) of biomass from class i was (Morchain et al., 2013, 2014)

(9) $$\frac{X_{i}}{\Delta \mu }\left(\frac{1}{T_{X}}+\mu_{i}\right)\left(Y_{XS}q_{S}\frac{S}{S+K_{S}}-\mu_{i}\right),$$

where $X_{i}$ is the biomass concentration in class i ($g\;L^{-1}$), Δμ the difference in specific growth rate between adjacent classes ($h^{-1}$), $T_{X}$ the time‐scale of growth rate adaptation ($h^{-1}$) as defined by Morchain and Fonade (2009) and Morchain et al. (2013), and $\mu_{i}$ the specific growth rate of class i ($h^{-1}$). The term $Y_{XS}q_{S}S∕\left(S+K_{S}\right)$ represents the equilibrium growth rate ($h^{-1}$) defined by the local environment (Morchain et al., 2013, 2014). With a positive adaptation rate (Equation 9), the class i's biomass was transferred to the class above (i+1) and with a negative rate to the class below (i−1). In total, the population tries to adapt toward the equilibrium growth rate (all of the biomass at the two classes closest to equilibrium), but the heterogeneity of local substrate concentrations results in a distribution of growth rates (Morchain et al., 2013, 2014).

The distribution of specific substrate uptake rate ($g\;g^{-1}\;h^{-1}$) was modeled similarly to the specific growth rate (Equation 9) but with classes $q_{i}=q_{S}\left(2i-1\right)∕20$ (i∈1,2,…,10), class discretization Δq ($g\;g^{-1}\;h^{-1}$), time constant $T_{S}$ ($h^{-1}$) as defined by Morchain et al. (2014), and equilibrium term $q_{S}S∕\left(S+K_{S}\right)$ ($g\;g^{-1}\;h^{-1}$). Further details of the population balance methodology can be found in the works of Morchain et al. (2013, 2014).

### 2.3 Software

Python 3.8.5 language (www.python.org) and the packages scipy 1.5.2 (Virtanen et al., 2020), numpy 1.19.2 (Harris et al., 2020), and pandas 1.1.3 (McKinney, 2010; The Pandas Development Team, 2020) were used for all calculations and simulations. Mixing times reported in the literature were recovered from the published figures with WebPlotDigitizer (Rohatgi, 2020).

### 2.4 Numerical methods

The initial value problems (mixing time and pH) were solved with the BDF‐method (backward differentiation formula) of the scipy.integrate module. The fed‐batch steady‐states (bioreaction) were solved by integration with the backward Euler method using constant time steps until the steady state was reached. The step vectors were solved from analytically calculated Jacobians with the stabilized biconjugate gradient algorithm (bicgstab in scipy.sparse.linalg). Ideal homogeneous reactor results were used as initial guesses for the large‐scale fed‐batch simulations.

## 3 THEORETICAL ASPECTS

### 3.1 1D diffusion equations

Feed point placement was analyzed separately in the axial, radial, and tangential dimensions by using 1D diffusion equations:

(10) $$\frac{\partial u}{\partial t}=d\frac{\partial^{2}u}{\partial z^{2}},$$

(11) $$\frac{\partial u}{\partial t}=d\left(\frac{1}{r}\frac{\partial u}{\partial r}+\frac{\partial^{2}u}{\partial r^{2}}\right),$$

(12) $$\frac{\partial u}{\partial t}=d\frac{1}{r^{2}}\frac{\partial^{2}u}{\partial \phi^{2}},$$

respectively, as models of macromixing. In Equations (10–12), u is the injected substance's dimensionless concentration normalized by the injected quantity to yield a spatial mean of 1, t time (s), d turbulent diffusivity ($m^{2}\;s^{-1}$) assumed to cover all forms of transport, z axial coordinate (m), r radial coordinate (m), and ϕ tangential coordinate. The domains were [0,H] for axial, [0,R] for radial, and [0,Φ] for tangential, where H is the height (m), R the radius (m), and Φ the cylindrical sector (0<Φ≤2π) of the modeled volume. Each of the three domains was insulated (zero‐gradient, symmetry) with no mass transfer across the boundaries. The axial and radial dimensions were solved with a point‐addition of feed at $z_{0}$ or $r_{0}$ as the initial condition. In general, the tangential dimension should be solved with a periodic boundary, but setting the feed pulse to the domain's middle and using the symmetry boundary conditions is equivalent and in the context of this work more convenient. Therefore, the tangential dimension was solved with closed boundaries and the feed pulse at $\phi_{0}=\Phi ∕2$. Only Φ=2π (whole cylinder) and integer fractions thereof are valid tangential domains owing to the symmetry boundaries.

The diffusion equations are analogous to transient heat conduction in insulated domains, and their solutions can be found, for example, in heat transfer textbooks (e.g., Cole & Beck, 2010). In each of the three dimensions, the time‐dependent concentration relative to equilibrium after a pulse addition of feed is a series, whose time‐dependent terms decay exponentially:

(13) $$u_{x}=1+\sum_{m=1}^{\infty }A_{xm}\text{exp}\left(-k_{xm}t\right).$$

The pre‐exponential terms $A_{xm}$ are

(14) $$A_{zm}=2\cos\left(m\pi \frac{z_{0}}{H}\right)\cos\left(m\pi \frac{z}{H}\right),$$

(15) $$A_{rm}=\frac{J_{0}\left(\beta_{m}r_{0}∕R\right)J_{0}\left(\beta_{m}r∕R\right)}{{\left(J_{0}\left(\beta_{m}\right)\right)}^{2}},$$

(16) $$A_{\phi m}=2\cos\left(m\pi \right)\cos\left(2m\pi \frac{\phi }{\Phi }\right),$$

in the axial, radial, and tangential dimensions, respectively. $J_{0}$ in Equation (15) is the zeroth‐order Bessel function of the first kind and $\beta_{m}$ is the mth root of the first‐order Bessel function of the first kind. The respective axial, radial, and tangential first‐order rate constants $k_{xm}$ are

(17) $$k_{zm}=m^{2}\pi^{2}\frac{d}{H^{2}},$$

(18) $$k_{rm}=\beta_{m}^{2}\frac{d}{R^{2}},$$

(19) $$k_{\phi m}=4m^{2}\pi^{2}\frac{d}{\Phi^{2}r^{2}}.$$

It is worth noting that the 1D tangential solution depends also on the radial coordinate through Equation (19). In general, the radial and tangential domains are interconnected, but they were studied separately here.

### 3.2 Axial placement of a single feed point

Given that the macromixing limitations in typical high aspect ratio bioreactors exist predominantly in the axial direction (Cronin et al., 1994; Vasconcelos et al., 1995), the optimal placement of a single feed point was first studied by analyzing the axial diffusion equation. The first term in the series (Equation 10) dominates the solution's long‐term behavior and thus the mixing time through $t_{95}\sim 1∕k_{z1}$ (Kawase, 1989), as the rest of the terms decay exponentially faster ($m^{2}$ in rate constants, Equation 17). The factor $\cos\left(m\pi z_{0}∕H\right)$ in the pre‐exponentials (Equation 14) suggests that placing the feed at the middle ($z_{0}∕H=0.5$) would remove the solution's first, rate‐limiting term as the relation cos(0.5mπ)=0 holds with all odd m. The solution's next limiting term would then be the second term (m=2) associated with a four‐fold rate constant $k_{z2}=2^{2}k_{z1}$. According to the model, placing the feed at the center $z_{0}=0.5H$ instead of the top $z_{0}=H$ (or bottom $z_{0}=0$) improves macromixing substantially by reducing the mixing time to a quarter of the original.

**Figure 2:**
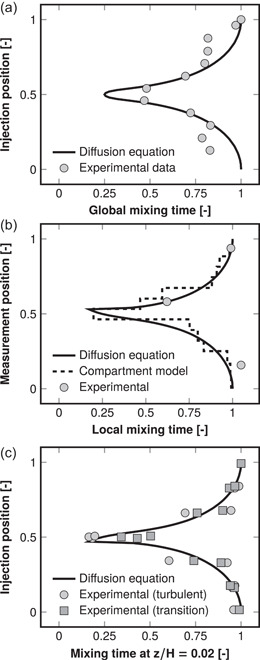
Effect of single feed point placement on mixing times. Both the experimental and modeled mixing time profiles have been normalized by the respective values found at the top (vertical axis 1) and the axial positions by the working height. Diffusion equation refers in each panel to the one‐dimensional axial diffusion equation defined in Equations (10), (13), (14), and (17). (a) The tracer injection point was varied and the global mixing time (Section 2.2.1) was measured. The 12 decolorization experiment data points are from a 600 L stirred tank with two impellers (Cronin et al., 1994). (b) The tracer injection point was kept at 94% of working height and the local mixing time was determined across the height. The three experimental data points are from an over 20 $m^{3}$ aerated tank stirred with four impellers (Vrábel et al., 1999) and the compartment model predictions are from the same study. (c) The measurement point was kept at 2% of working height and the local mixing time was measured as a function of the tracer injection position. The 13 experimental data points are from a 60 L stirred tank with three impellers both in turbulent and transition (impeller Reynolds number ${\;\text{Re}}_{\text{i}\;}=250$) flow regimes (Alves et al., 1997).

Figure 2 shows how the feed point's position along a reactor's working height affects macromixing according to the 1D axial diffusion equation. Three cases were considered with experimental data available for comparison: (1) The effect of feed location on global mixing time defined in Section 2.2.1 (Cronin et al., 1994). (2) The effect of measurement position on locally measured mixing time while injecting the tracer at the top (Vrábel et al., 1999). Local measurement and injection positions are interchangeable (z and $z_{0}$ in Equation 14). (3) The effect of injection position in both turbulent and transition (impeller Reynolds number ${\;\text{Re}}_{\text{i}\;}=250$) regimes while measuring the mixing time at the bottom (Alves et al., 1997). The cited experiments covered in total 28 injection or measurement positions in working volumes ranging from 59 L to over 20 $m^{3}$ stirred with two to four impellers. The experiments suggested that the center ($z_{0}=0.5H$) is superior to the top or bottom. The rest of the injection positions resulted in a funnel‐like distribution of mixing times around the optimal center. A similar axial distribution was predicted by the diffusion equation as well. Owing to its simple and spatially homogeneous nature, the model was incapable of predicting the asymmetry that was present in some of the experimental data, which was due to the stagnant flow close to the liquid surface (Cronin et al., 1994). The good agreement found between the predictions and literature data suggests that the simple diffusion equations could be used to predict the effect of feed placement.

### 3.3 Axial placement of multiple feed points

**Figure 3:**
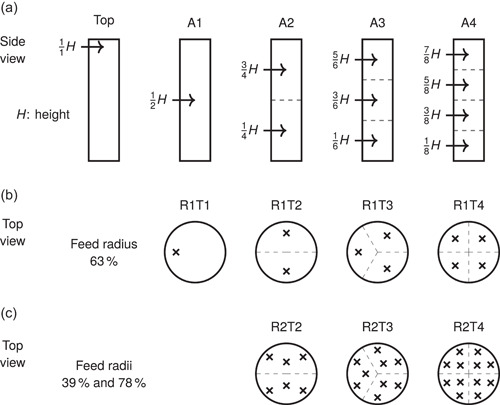
Optimal placement of multiple feed points. (a) Axial domain should be divided (dashed lines) into equal subdomains with a centrally placed feed point in each. The arrows represent the feed positions relative to the liquid height. (b) Given one radial coordinate the feed points should be placed symmetrically across the perimeter of 63% radius. (c) With two radial coordinates, the inner 39% radius receives one symmetrically placed feed point per subdivision and the outer 78% radius two symmetrically placed feed points per subdivision. A feed arrangement A*x*R*y*T*z* is composed of the radial‐tangential pattern RyTz at every axial position of Ax.

Motivated by both the marked potential of correct feed placement in reducing the mixing time and the predictive power of the axial diffusion equation, the axial placement of multiple feed points was analyzed next. As the first‐order rate constants of the axial equation are inversely proportional to the reactor's working height squared, $H^{2}$, any reduction in height would greatly increase the mixing rate (i.e., decrease the mixing time) if the turbulent diffusivity d remained constant ($k_{zm}\sim d∕H^{2}$, Equation 17). Likewise, increasing the diffusivity would improve the rate in a given geometry. However, decreasing the actual working height is impractical and a greater diffusivity would require a higher power input (Kawase, 1989). How could this square relationship then be exploited without changing the reactor's geometry or the stirrer's power? The symmetry or zero‐gradient boundaries of the model equation (Section 3.1) imply that placing N feed points symmetrically across the whole height at axial positions of

(20) $$\frac{z_{i}}{H}=\frac{2i-1}{2N}i=1,2,\text{…},N,$$

divides the working height into N equally sized compartments having a height of H∕N each. As the height is replaced by H∕N in Equation (17), the limiting rate constant increases in proportion to $N^{2}$: two symmetrically placed feed points multiply the mixing rate by 4 and three points by 9. Compared with a single feed at the top, four ideally placed feed points multiply the rate in theory by 64 ($4\times 4^{2}$). Figure 3a illustrates the optimal axial placement of feed points (Equation 20). Interestingly, a similar symmetry is often used in the placement of multiple impellers.

### 3.4 Radial‐tangential placement

In bubble columns and lower aspect ratio single‐impeller stirred tanks, the radial and tangential dimensions may also limit the overall rate of mixing. As with the axial dimension, the respective 1D diffusion equations reveal optimal feed placement in the radial‐tangential plane. Both the use of (1) only one or (2) two or more radial coordinates need to be considered.

For case 1 (single radial coordinate), the optimal radial‐tangential placement is found analogously to the single‐ and multipoint axial placements: The first (m=1) radial term is eliminated by finding the radial feed point that satisfies the condition

(21) $$J_{0}\left(\beta_{1}\frac{r_{0}}{R}\right)=0,$$

which yields $r_{0}∕R\approx 0.628$ (see Equation 15). The new limiting radial term (m=2) is then associated with a ${\left(\beta_{2}∕\beta_{1}\right)}^{2}\approx 3.35$ fold rate. Placement of multiple feed points in the tangential dimension behaves in the same way as in the axial dimension: placing N feed points symmetrically across the whole tangential domain divides the effective domain size by N and multiplies the limiting rate constant by $N^{2}$. As conclusion, the feed points should be placed symmetrically at approximately 63% radius around the whole reactor. Figure 3b illustrates the optimal radial‐tangential placement of feed points with one radial coordinate.

Considering two or more radial coordinates (case 2), the radial pre‐exponentials (Equation 15) have the useful property that the Nth pre‐exponential has exactly N roots at the interval $r_{0}\in \left[0,R\right]$. The Nth radial term remains zero when the N radial feed coordinates $r_{i}$ satisfy the condition

(22) $$J_{0}\left(\beta_{N}\frac{r_{i}}{R}\right)=0,i=1,2,\text{…},N,$$

which yields optimal feed coordinates $r_{i}$ at

(23) $$\frac{r_{i}}{R}=\frac{\alpha_{i}}{\beta_{N}},$$

where $\alpha_{i}$ is the ith root of $J_{0}$. The remaining limiting lower‐order terms (m<N) are then eliminated by assigning the radial feed points $r_{i}$ appropriate weights $w_{i}$, which are found from the linear system of equations

(24) $$\left[\begin{array}{cccc} J_{0}\left(\beta_{1}r_{1}∕R\right) & J_{0}\left(\beta_{1}r_{2}∕R\right) & \text{⋯}\; & J_{0}\left(\beta_{1}r_{N}∕R\right)\\ J_{0}\left(\beta_{2}r_{1}∕R\right) & J_{0}\left(\beta_{2}r_{2}∕R\right) & \text{⋯}\; & J_{0}\left(\beta_{2}r_{N}∕R\right)\\ \vdots & \vdots & \ddots & \vdots \\ J_{0}\left(\beta_{N-1}r_{1}∕R\right) & J_{0}\left(\beta_{N-1}r_{2}∕R\right) & \text{⋯}\; & J_{0}\left(\beta_{N-1}r_{N}∕R\right)\\ 1 & 1 & 1 & 1 \end{array}\right]\left[\begin{array}{c} w_{1}\\ w_{2}\\ \vdots \\ w_{N} \end{array}\right]=\left[\begin{array}{c} 0\\ 0\\ \vdots \\ 1 \end{array}\right].$$

With two feed points, the optimal feed coordinates are 34.4% and 78.7% of radius weighted in proportions of approximately 1:2.31. The weighting can be implemented by using multiple tangential coordinates with equal flow rates on a given radial coordinate or by setting nonuniform feed flow rates in proportion to the weights. However, to retain equal tangential rate constants in the different radial positions, the outer radial coordinates require more feed points than the inner ones to keep equal perimeters Φr in Equation (19). Equal rate constants are preserved if the number of feed points at the different radii is proportional to the radii themselves. For example, a radius of 2 units would require twice the number of tangential feed points used at a radius of 1 unit. Satisfying the optimum criteria for both the radial and tangential dimensions simultaneously is difficult, but a reasonable compromise can be obtained by rounding the radial coordinates close to the actual optima such that integer ratios are obtained. As conclusion, the feed points ought to be placed at concentric circles around the reactor. The circle radii are to have integer ratios and the number of symmetrically placed feed points in the circles are to follow the same integer ratios. Figure 3c illustrates a near‐optimal radial‐tangential placement with two radial coordinates 39% and 78% of radius, which have been rounded from the optimal 34.3% and 78.7%.

## 4 RESULTS

The large‐scale bioreactor simulations were carried out with a single top feed for reference and with nine optimal (Sections 3.2, 3.3, 3.4) feed arrangements A*x*R*y*T*z*, where *x*, *y*, and *z* refer to the number of axial, radial, and tangential coordinates, respectively. 1, 2, or 4 axial coordinates (A1, A2, and A4, respectively) with a single feed point (R1T1), two feed points (R1T2), or six feed points (R2T2) each were used in the optimal feed arrangements, which are illustrated in Figure 3.

### 4.1 Mixing

**Figure 4:**
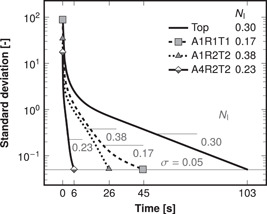
Standard deviation of dimensionless tracer concentration in reactor B13 after tracer pulse with top feed and various optimal multipoint feeds. The feed arrangements A*x*R*y*T*z* contain *x* axial, *y* radial, and *z* tangential coordinates (Figure 3). The 5% line is the threshold for mixing time. The horizontal lines represent the inhomogeneity numbers $N_{\;\text{I}\;}$ (median standard deviations in the mixing time interval). Note the logarithmic scaling of the vertical axis.

The mixing times and inhomogeneity numbers resulting from all tracer simulations are compiled in Table 3. As an illustration of the improvements attained by optimal feeds, Figure 4 shows both the time evolution of the tracer concentration's standard deviation and the inhomogeneity numbers (median standard deviation in the mixing time interval) in reactor B13 with representative feed arrangements. The time‐evolution of standard deviation was similar also in the other reactors (Supporting Information: Figure S1). With the typical single feed at the top the simulated mixing times were over 100 s in each of the four reactors. The mixing times were reduced to less than half of the original in reactors R4 and B13 when the single feed point was placed at the middle height (feed arrangement A1R1T1). In R4, the central placement resulted in a four‐fold mixing rate (mixing time a quarter of the original) as predicted in Section 3.2, but the B13's slightly over two‐fold rate did not reach the prediction. In reactors R1 and B6, the mixing rates relative to the top feed were modest at 1.2‐ and 1.1‐fold, respectively. However, the central placement reduced the inhomogeneity numbers at least to 85% and at best to 57% relative to the top feed in reactors R1, B13, and B6 with the under four‐fold mixing rates.

**Table 3:** Mixing times and inhomogeneity numbers in reactors R4, R1, B13, and B6 with top feed and optimal multipoint feeds

	R4		R1		B13		B6	
Feed	$t_{95}$/s	$N_{\;\text{I}\;}$	$t_{95}$/s	$N_{\;\text{I}\;}$	$t_{95}$/s	$N_{\;\text{I}\;}$	$t_{95}$/s	$N_{\;\text{I}\;}$
Top	154	0.263	141	0.404	103	0.297	135	0.387
A1R1T1	36.4	0.268	120	0.343	45.2	0.169	122	0.307
A1R1T2	36.5	0.267	97.3	0.206	45.1	0.150	58.0	0.344
A1R2T2	36.5	0.272	88.5	0.188	26.0	0.380	49.4	0.207
A2R1T1	13.6	0.219	109	0.289	31.4	0.188	123	0.309
A2R1T2	12.9	0.127	77.5	0.196	28.8	0.087	52.9	0.301
A2R2T2	9.01	0.250	55.8	0.172	12.8	0.259	28.5	0.208
A4R1T1	16.9	0.145	110	0.294	29.2	0.205	122	0.307
A4R1T2	16.6	0.090	77.8	0.183	23.9	0.077	52.5	0.295
A4R2T2	17.8	0.103	64.5	0.182	6.36	0.225	26.2	0.188

*Note*: The feed arrangements A*x*R*y*T*z* contain *x* axial, *y* radial, and *z* tangential coordinates (Figure 3). Symbols: $t_{95}$, mixing time; $N_{\;\text{I}\;}$, inhomogeneity number.

Upon employing multiple feed points at the middle height (A1R2T2), mixing in the high aspect ratio bubble column B13 reached the predicted four‐fold rate relative to the top feed. Similarly to B13, the lower aspect‐ratio bubble column B6 was mixed with an almost three‐fold rate. The mixing rate in reactor R1 increased to 1.6‐fold relative to the top feed. The feed arrangement A1R2T2 reduced the inhomogeneity numbers to 47% and 53% in reactors R1 and B6, respectively, where the predicted four‐fold rate was not met.

Incrementing axial feed coordinates (feed arrangements A2RyTz and A4RyTz) diminished the mixing times further in each reactor. The absolute improvements were considerable: considering the top feed as a starting point, the mixing time was reduced by more than a minute in each reactor. The multi‐impeller stirred tank R4 achieved even the 16‐fold mixing rate predicted in Section 3.3 with two axial coordinates (A2R2T2), but in the other reactors, the relative improvements did not meet the prediction. Surprisingly four axial coordinates (A4RyTz) resulted in longer mixing times than two (A2RyTz) in R4. A substantial 16‐fold mixing rate relative to the top feed was attained also in B13 with the feed arrangement A4R2T2. The inhomogeneity numbers were reduced further in each reactor when multiple axial coordinates were utilized. With the A4RyTz feed arrangements, the inhomogeneity numbers were diminished even down to 34%, 45%, 26%, and 49% in reactors R4, R1, B13, and B6, respectively.

### 4.2 Concentration gradients

All pH‐control and bioreaction simulation results with 10 $g\;L^{-1}$ biomass concentration are compiled in Table B1. Similarly to the mixing time simulations, the addition of feed points resulted in more homogeneous behavior of the reactors. The volumetric standard deviation of each simulated variable or quantity was reduced, and the volumetric means approached the value found in an ideal homogeneous reactor. With multiple feed points, the reactors R4 and B13 approximated the ideal homogeneous reactor more closely than the reactors R1 and B6.

**Figure 5:**
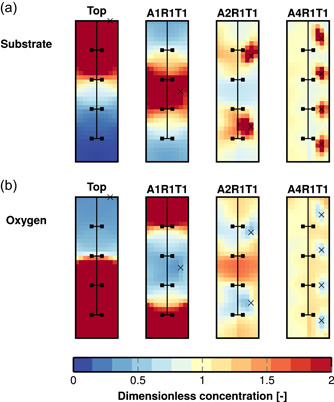
Simulated concentration of substrate (a) and dissolved oxygen (b) in reactor R4 with 10 g L^−1^ biomass concentration and a 4 $g\;L^{-1}\;h^{-1}$ substrate feed rate at the top or through an optimal multipoint feed. The concentrations have been normalized by respective concentrations (substrate 16.7 $\text{mg}\;L^{-1}$, oxygen 0.113 $\text{mg}\;L^{-1}$) calculated in an ideal homogeneous reactor at the same conditions. Note the color scale limits (values above twice the ideal reactor value are shown as 2). The feed arrangements AxR1T1 contain x axially distributed feed points (Figure 3).

Considerable gradients in pH and substrate and dissolved oxygen concentrations were found in each reactor when the feed was at the top. The pH and concentration of substrate was always the highest close to the top feed point and lowest at the bottom of the reactor. In contrast, the concentration of dissolved oxygen was lowest in the top feed zone and highest away from it. Multiple feed points displayed the same patterns: pH and substrate concentration remained the highest and dissolved oxygen concentration the lowest in the vicinity of the feed points. However, both the highs and lows in pH and concentrations were brought closer to the mean when multiple feed points were in use. Figure 5 illustrates this by showing the substrate and oxygen gradients and how they were mitigated by the addition of feed points in reactor R4. The substrate gradients were of similar nature in the other reactors as well (Supporting Information: Figures S2–S4), and the pH gradients 10 s after the carbonate pulse (Supporting Information: Figure S5) were similar to the substrate gradients. In each reactor, the magnitude of the gradients seemed to correlate with the mixing times and standard deviations shown in Tables 3 and B1, respectively. In reactors R4 and B13, there were virtually no gradients of substrate, oxygen, and pH left with the multipoint feed arrangements A2R2T2 and A4R2T2. In reactors R1 and B6, the gradients were not entirely removed by the multipoint feeds, but their magnitude was reduced.

**Figure 6:**
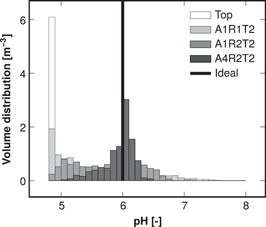
Distribution of pH in reactor R1 10 s after pH‐correcting pulse of carbonate in a 100 $\text{mmol}\;L^{-1}$ carbonate buffer solution initially at a pH of 4.8. Responses to top and optimal multipoint pulses are shown. Ideal homogeneous reactor behavior is shown for reference (whole volume has pH 6). The feed arrangements A*x*R*y*T*z* contain *x* axial, *y* radial, and *z* tangential coordinates (Figure 3).

**Figure 7:**
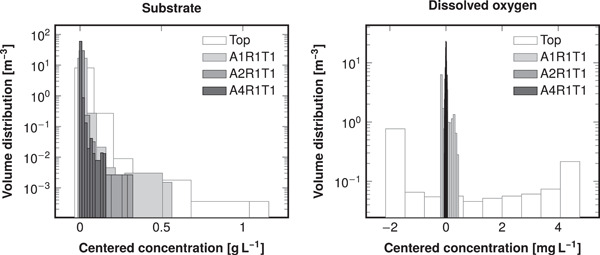
Distribution of substrate (left) and dissolved oxygen (right) in reactor R4 with 10 $g\;L^{-1}$ biomass concentration and a 4 $g\;L^{-1}\;h^{-1}$ substrate feed rate at the top or through an optimal multipoint feed. The substrate distributions were similar in the other reactors as well (not shown, for visual comparison, see Figures 5 and Supporting Information: Figures S2–S4). The concentrations have been centered by respective mean concentrations such that the mean of each shown distribution is 0. The feed arrangements A*x*R*y*T*z* contain *x* axial, *y* radial, and *z* tangential coordinates (see Figure 3). Note the logarithmic scaling of the vertical axis.

Figure 6 shows the volume distribution of pH in the reactor R1 10 s after pH correction pulse, and the distributions were similar in the other reactors (Supporting Information: Figure S6). The volume distributions of substrate and dissolved oxygen concentration were similar (Figure 7) to the pH distributions: the top feed resulted in broad distributions centered relatively far away from the ideals, and multiple feed points narrowed the distributions and centered them closer to the ideals. The spread (broadness) of the volume distributions is quantified by the volumetric standard deviations shown in Table B1. Likewise, the deviation of volumetric means from the ideal reactor values in Table B1 represents how far from the ideal the distributions were centered.

The bioreaction simulations were carried out also with a higher, $X=50\;g\;L^{-1}$, biomass concentration, and the results are compiled in Supporting Information: Table S1. Overall the gradients were steeper in each case than with the lower 10 $g\;L^{-1}$ biomass concentration, and similarly, the use of multiple optimally placed feed points mitigated the gradients substantially (Supporting Information: Figure S7). Interestingly, the substrate gradients were so severe in each reactor with the top feed that the overall biomass yield was negative, which indicates that the simulated 50 $g\;L^{-1}$ biomass was higher than what would actually have been achievable with the given biomass yield definition (Equation 8). In reactors R4 and B13, the feed arrangement A4T2T2 recovered a positive biomass yield and thus the feasibility of the 50 $g\;L^{-1}$ biomass concentration (Supporting Information: Table S1).

### 4.3 Adaptation of biomass

**Figure 8:**
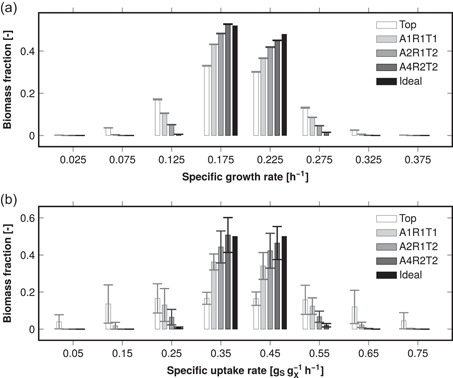
Simulated distribution of biomass‐specific growth (a) and substrate uptake (b) rates in reactor B13 with 10 $g\;L^{-1}$ biomass concentration and a 4 $g\;L^{-1}\;h^{-1}$ substrate feed at the top or through an optimal multipoint feed. The two highest classes with only negligible biomass are not shown. The error bars represent volumetric standard deviations. The feed arrangements A*x*R*y*T*z* contain *x* axial, *y* radial, and *z* tangential coordinates (Figure 3). The ideal homogeneous reactor results are shown for reference.

Figure 8 shows representative distributions of biomass‐specific growth and substrate uptake rates simulated in reactor B13 with a 10 $g\;L^{-1}$ biomass concentration. The results were similar in the other reactors as well (Supporting Information: Figures S8–S10). The top feed produced broad specific growth and uptake rate distributions (biomass distributed to several classes), but the use of multiple feed points narrowed them toward the ideal (biomass in two classes). The specific growth rate's distribution was spatially homogeneous even with the top feed with only minimal volumetric standard deviation in the biomass classes. In contrast, the uptake rate's distribution was spatially heterogeneous even when multiple feed points were used. However, the standard deviations relative to the respective means were decreased by the use of multiple feed points. The spread of the distributions correlated with the standard deviations shown in Table B1. With the higher $X=50\;g\;L^{-1}$ biomass concentration, the trends were the same but the distributions were centered to classes with lower specific rates (Supporting Information: Figure S11). It should be remembered that the equilibrium growth rates used in determining the growth rate distributions were calculated with a constant biomass yield (no biomass decay).

## 5 DISCUSSION

### 5.1 Mixing

Notable improvements in bioreactor mixing were realized in the simulations with the feed arrangements derived in Section 3. Both R4 and B13 achieved mixing times of 10 s (Table 3), which are common in laboratory‐scale reactors (Delafosse et al., 2014). In each reactor, the mixing rates were more than doubled (mixing time half of the original) relative to the common top feed setting. These reductions in mixing time can be given context by considering alternative ways to achieve them. Single‐ and multi‐impeller stirred tank and bubble column mixing time correlations state that the mixing time is related to specific power input by $t_{95}\propto {\left(P∕\left(\rho V\right)\right)}^{-1∕3}$ (Kawase, 1989; Magelli et al., 2013). A doubled mixing rate would require an 8‐fold specific power, and the over 10‐fold improvements simulated in R4 and B13 would demand infeasible, over 1000‐fold power inputs. Another way to highlight the effect of feed point number and placement is to consider how much smaller a volume would have the same mixing time with the same specific power without feed optimization. The same correlations imply that under constant specific power, the mixing time is related to reactor diameter by $t_{95}\propto T^{2∕3}$ and thus to volume by $t_{95}\propto V^{2∕9}$. The more than doubled mixing rates achieved in each reactor would then demand an over 20‐fold reduction in volume, and the 10‐fold mixing rates observed in R4 and B13 correspond to mixing $10^{-9∕2}\approx 1∕31600$‐fold volumes. As far as the macromixing time is concerned, the optimal feed arrangements figuratively scaled the simulated large‐scale reactors down to a laboratory scale. Furthermore, the inhomogeneity numbers were overall reduced in each reactor as the number of feed points was increased, which implies a lesser degree of heterogeneity in the reactor during the mixing time interval even when the mixing times were not considerably shortened by the addition of feed points.

It is necessary to remember, though, that the simulated mixing time improvements may have been greater than what could be obtained in practice, but experiments in pneumatically agitated reactors have confirmed that 2.9‐ to 6.6‐fold mixing rates are feasible by appropriate placement of only two feed points (Fu et al., 2005). For comparison, the feed arrangements A1R1T2 and A2R1T1 with two feed points achieved 2.2‐ and 3.2‐fold rates, respectively, in the bubble column B13. Another point of concern might be that the simulations were based on models that have been validated with water as the working fluid instead of viscous and potentially non‐Newtonian fermentation broths. In general, the flow conditions in a bioreactor may enter the transition regime between laminar and turbulent flow owing to the broth's viscosity. However, as was shown in Figure 2c, the axial placement of a single feed point affects mixing in a viscous model fluid in transition regime (${\;\text{Re}}_{\text{i}\;}=250$) in almost the same way as in water in turbulent regime (Alves et al., 1997). The experimentally observed trend of axial placement was similar to the diffusion model's prediction in both cases. Similarly, a 1D compartment model structure (discretization of a 1D diffusion equation) can describe multi‐impeller bioreactor mixing even in the transition regime with ${\;\text{Re}}_{\text{i}\;}$ down to 400 (Vasconcelos et al., 1996). Considering also that the main resistance to mixing in multi‐impeller bioreactors occurs at the boundaries between impeller regions (Cronin et al., 1994; Vasconcelos et al., 1995), where the exchange flow begins to diminish markedly as ${\;\text{Re}}_{\text{i}\;}$ decreases below 10,000 (Vasconcelos et al., 1996), the use of multiple axial feed coordinates can be expected to be highly beneficial also in the transition regime relevant to many fermentations as the main axial flow barriers are circumvented. Given that mixing in real fermentation broths is relatively little studied and quantified, some uncertainty with respect to actual fermentations persists when using models validated with model fluids. This same concern would have applied equally for hydrodynamically more sophisticated, CFD‐based simulations as well.

Some of the feed arrangements performed as predicted in Section 3, but others fell short of the ambitious expectations. The bubble column B13's initial deviation from A1R1T1 (single point at the middle height) prediction was understandable since the axial diffusion model (Equation 10) assumed homogeneity in the radial and tangential dimensions, and bubble columns with substantial gas flow are generally rather heterogeneous radially (Degaleesan et al., 1997). With multiple feed points at the middle height (A1R2T2), the assumed radial‐tangential homogeneity was re‐established sufficiently and B13 was mixed with the expected four‐fold rate. The B6 bubble column did not quite achieve the four‐fold rate, though, which was likely due to the column's large diameter (3.7 m) and cross‐section's insufficient coverage by the studied radial‐tangential placements. Adding further radial or tangential feed coordinates might have resulted in the expected four‐fold rate. In the bubble columns (B13 and B6), a large circulation current spanned the whole vessel contributing to deviations from the 1D diffusion models. Furthermore, the feed placement derivation could not account for the uneven distribution of liquid and gas within the bubble columns. It seems, therefore, that two or more radial and tangential coordinates should be used in both bubble columns and stirred tanks with high gas flow rates leading to impeller flooding and bubble column resembling flow fields (Alves & Vasconcelos, 1995; Machon & Jahoda, 2000). The R1's discrepancy between prediction and simulation is readily explained by the reactor's configuration: in a single‐impeller vessel with a low aspect ratio, the overall mixing is not limited in the same way by axial exchange between impeller regions like in high aspect ratio multi‐impeller vessels. Consequently, the simple diffusion equation cannot be as predictive for the single‐impeller vessel than for multi‐impeller ones. Reactor R4 attained the predicted 4‐ and 16‐fold rates with one and two axial feed coordinates, respectively, but fell short of the expectations thereafter. The axial diffusion equation with a single, global diffusivity parameter is most applicable at modeling a reactor as a whole. Therefore, as the number of axial feed points is increased, the equation is applied at smaller spatial scales where its suitability in modeling the reactor is eventually lost as it cannot include the effects of local advective flows or local differences in turbulence. Despite the fact that the ambitious predictions regarding the number of axial feed coordinates ($t_{95}\sim N^{-2}$) were not entirely met, the presented results imply that large‐scale bioreactors could be homogenized effectively by employing the proposed multipoint feeds.

### 5.2 Reactor performance

Performing a Taylor‐expansion and spatial‐averaging on the concave Monod‐type substrate uptake expression leads to (Pulkkinen & Metzler, 2015)

(25) $$\left\langle \frac{S}{S+K_{S}}\right\rangle =\frac{\langle S\rangle }{\langle S\rangle +K_{S}}-\frac{K_{S}\sigma_{S}^{2}}{{\left(\langle S\rangle +K_{S}\right)}^{3}}$$

where the spatial averaging is denoted by angle brackets 〈〉 and the substrate concentration's volumetric standard deviation by $\sigma_{S}$. Equation (25) shows that heterogeneity ($\sigma_{S}>0$) in substrate concentration leads to a decrease in the overall substrate consumption rate, all else being equal. Therefore, more efficient mixing has the potential to increase the rate of Monod‐type reactions. As the overall substrate consumption was 4 $g\;L^{-1}\;h^{-1}$ (equal to feed rate) by definition in these fed‐batch snap‐shot simulations, Equation (25) implies that an increase in substrate concentration's standard deviation caused by the competition between reaction and mixing necessitates a corresponding increase in the reaction's driving force, the substrate's mean concentration. This trend is apparent in Table B1 and Supporting Information: Table S1: lower substrate concentration means were associated with lower standard deviations, and the same can be observed in the simultaneous decrease of both the mean and standard deviation of the substrate uptake's timescale. Similarly, higher mean dissolved oxygen concentration with top feeding was due to suboptimal capability to consume oxygen. Figure 5B and Supporting Information: Figure S8 reveal that with unoptimal top feeding, the concentration of oxygen was high only where substrate was limiting. Vice versa, oxygen concentrations were low where the substrate was abundant, that is, close to the feed. Volumetric means of substrate concentration, substrate consumption time‐scale, and dissolved oxygen concentration were brought close to ideal homogeneous reactor values, which translates to the removal of the reaction mixing competition and restoration of ideal reactor performance in large reactors. Furthermore, the oxygen consumption capacity was improved by the more effective homogenization. With a higher $50\;g\;L^{-1}$ biomass concentration, the substrate and oxygen consumptions' time‐scales were lower and correspondingly, the competition between reaction, oxygen transfer, and mixing was more severe (Supporting Information: Table S1 and Figure S7). However, the volumetric means were still brought close to ideal by the proposed multipoint feeds even though a higher degree of heterogeneity remained (Supporting Information: Table S1). It needs to be remembered, though, that the simulation method addressed only oxygen heterogeneity caused by the bioreaction. Other heterogeneity‐contributing factors, that is, local differences in gas holdup, gas‐phase concentrations, mass transfer coefficient, and hydrostatic pressure, were deliberately omitted to isolate the effect of substrate feeding. The simulations showed that the reaction's contribution to oxygen heterogeneity was removed by appropriate feeding.

Yield improvements with baker's yeast have been reported when feed points were added (Hansford & Humphrey, 1966) to a stirred tank reactor and when a single feed point was placed close to an impeller (Dunlop & Ye, 1990). In the $X=10\;g\;L^{-1}$ simulations reported here, the ideal homogeneous reactor biomass yield was restored by the addition of feed points. In the $X=50\;g\;L^{-1}$ simulations, the yields did not reach the ideal reactor values, but they were improved considerably nevertheless in each case. In reactors R4 and B13, a positive biomass yield was re‐established (Supporting Information: Table S1) by the A4R2T2 feed arrangement, which indicated that the appropriate addition of feed points could increase the upper limit of biomass concentration that is achievable in a given reactor. Unlike in the referenced experiments, in the simulations presented here, the yield improvements were entirely due to macromixing improvements as micromixing was not modeled. However, more efficient macromixing should also enhance micromixing. Local mass‐transfer preceding the substrate uptake by the cells is proportional to the difference between the bulk concentration and the local concentrations within the smallest turbulent eddies (Dunlop & Ye, 1990). Analogously to the pH distribution shown in Figure 6, insufficient mixing associated with the conventional top feed leads to substrate depletion in a large fraction of the total volume as is visualized in Figures 5A and Supporting Information: Figure S7. Regions with low bulk concentration are bound to suffer micromixing limitations as the driving force of mass‐transfer, the concentration difference, is already exhausted. Restoring a homogeneous macroscale concentration field can then be expected to maintain the necessary, reaction‐driving concentration difference between the bulk and the local. In stirred tanks, the micromixing time‐scales can be further optimized by bringing the feed points closer to the impellers (Dunlop & Ye, 1990).

### 5.3 Physiological consequences

One of the most often mentioned problems in bioreactor scale‐up is the acetate production with *E. coli* (Eiteman & Altman, 2006; Enfors et al., 2001; Xu et al., 1999) due to anaerobic mixed acid fermentation, aerobic overflow metabolism, or both (Xu et al., 1999). The mechanisms behind both of these are related: too high a substrate concentration and uptake rate can exceed either the local oxygen availability or the cell's oxidative capacity. Accumulation of formate, a product of anaerobic metabolism, revealed the presence of anoxic regions in experiments conducted in the R4 reactor (Xu et al., 1999). Similarly, the simulated high substrate concentrations close to the top feed created locally high demands of oxygen, which resulted in oxygen depletion in the R4's upper part (Figures 5 and Supporting Information: Figure S7). Though not simulated here, the high substrate concentrations found close to the top feed could lead also to aerobic overflow (Szenk et al., 2017). Similar side‐formation of ethanol is known with *Saccharomyces cerevisiae*. As could be anticipated, the simulations suggested two benefits in multipoint feeding: (1) High substrate concentrations were avoided, which should aid in preventing aerobic overflow. (2) Oxygen depletion was avoided, which ought to prevent anaerobic metabolism. Appropriate feeding may then aid in accomplishing one of the goals of fed‐batch operations: avoidance of high substrate concentrations, which result in side‐product formation and oxygen limitations.

Extremes of substrate, dissolved oxygen, dissolved carbon dioxide concentration, pH (Amanullah et al., 2001; Langheinrich & Nienow, 1999), and temperature, along other factors may stress the host organism and influence product formation. Microbial stress responses are activated when the cells are exposed to overly high concentration of substrate in the feed zone (Enfors et al., 2001; Schweder et al., 1999). In the pH control simulations, a small but nontrivial portion of the reactor's volume remained at a relatively high pH even 10 s after the pulse. Experimentally in the 8 $m^{3}$ R1 reactor, the addition of carbonate resulted in 0.6 unit pH excursions above the targeted value (Langheinrich & Nienow, 1999), which resembles what was simulated here. However, the experiments cannot be directly compared to the simulations owing to the different initial pH and buffer concentrations. Based on the simulation results, multipoint feeding should reduce the stress responses caused by poor mixing.

Cultivation history influences the culture's response to an excess of substrate or the presence of alternative substrates such as organic acid side‐products (Brand et al., 2018; Enjalbert, 2015). Unlike chemical catalysts, cells monitor their surroundings and adjust themselves continuously. In a heterogeneous large‐scale bioreactor, this leads to unnecessary back‐and‐forth switching of gene expression as the cells are constantly being exposed to different environments (Enfors et al., 2001; Schweder et al., 1999). Another consequence is that the population does not necessarily have the time to adapt to the changing conditions, which results in heterogeneity in the reactor's biological phase, the population of cells. Similarly to earlier simulations with the population balance methodology, both the growth and substrate uptake rate distributions were broad in a heterogeneous reactor (Morchain et al., 2014). Figures 8 and Supporting Information: Figures S8–S11 show how the efficient homogenization of the liquid phase also yielded a more homogeneously‐responding population. It should be noted that the simulations presented here were conducted with a substrate concentration field that was not influenced by the population balance (one‐way coupling as used by Pigou & Morchain, 2015). Including the effects of the population's substrate uptake rate adaptation on the substrate concentration field would have been more correct (Morchain et al., 2014), but due to the relatively low adaptation time‐scale of the uptake rate this was not strictly necessary.

### 5.4 Bioreactor control

Usually, the pH and dissolved oxygen tension within a bioreactor are measured by single or at most a few probes. With the typical top feed both of these quantities are expected to be quite heterogeneous in a large‐scale reactor, which also has experimental evidence (Langheinrich & Nienow, 1999; Oosterhuis & Kossen, 1984). In such a situation, the sensors are no longer representative of the reactor as a whole. Indeed, in a large‐scale aerobic *E. coli* fermentation experiment conducted in reactor R4, the two oxygen probes showed no oxygen limitations even though the measured formate accumulation implied that approximately 11% of the reactor should have been anoxic (Xu et al., 1999). The $X=10\;g\;L^{-1}$ simulations (Table B1) with a top feed showed a relative standard deviation of 119% in dissolved oxygen concentration, whereas the multipoint feed A4R2T2 reduced the relative standard deviation even down to 8%. It is easy to observe in Figures 5B and Supporting Information: Figure S8B that a single oxygen tension probe cannot represent the whole reactor with the top and A1R1T1 feeds, whereas with the multipoint feeds, the whole reactor could be represented by a single probe quite reliably. Likewise a point‐measurement of pH in a heterogeneous reactor might lead to faulty pH‐control, where the control is activated or deactivated too soon or too late as the pH probe represents only its immediate vicinity (Langheinrich & Nienow, 1999). Table B1 shows that 10 s after the correcting pulse with the top feeding, the mean pH was over 0.5 pH units below the target in each reactor even though the carbonate pulse was exactly the amount required to achieve the desired pH of 6. Such a situation could lead to overapplication of pH correctives unless the delay caused by mixing is properly accounted for by the control algorithm or by multiple probes (Langheinrich & Nienow, 1999). With multipoint feeds A2R2T2 and A4R2T2, the mean pH after 10 s was within 0.06 pH units of target even in the slowly mixing R1 reactor. Such a situation is less prone to overdosage of correctives. Appropriate multipoint feeds should then result in more precise and efficient reactor control as the sensors would represent the actual conditions within the reactor more reliably across the whole working volume.

### 5.5 Implementation

The diffusion model, these simulations as well as intuition suggest that distributing the feed evenly throughout the whole volume should lead to most efficient mixing. This is in accordance with the suggestions of placing an inlet at each impeller or to multiple well‐mixed zones (Cronin et al., 1994; Enfors et al., 2001; Fowler & Dunlop, 1989; Larsson et al., 1996). Extending the diffusion equation‐derived feed placements shown in Figure 3 would eventually lead to a homogeneous and full coverage of the entire working volume. A simple way to achieve a thorough distribution of feed into the whole volume might be based on, for example, perforated rods or a perforated coil analogous to heat transfer equipment. Simulations and experiments should establish, whether such a feed arrangement would be feasible in bioreactors. Figure 2 showed that the placement of a single feed point is rather sensitive, but distributing the feed as evenly throughout the volume as possible ought to overcome this sensitivity.

Two concerns, biological contamination and engineering complications, are easily raised upon considering multipoint feeds. Depending on the nature of the product, contamination may result in the failure of up to 17% of fermentations (Morandi & Valeri, 1988). Processes involving slowly growing host organisms are particularly susceptible. The extra internals required by a multipoint feed would hamper mixing and mass and heat transfer to some extent, but the severeness of this would probably be comparable to that of heat transfer internals. Hydraulic losses within the feed pipes are likely to be manageable, as in fermentations, the feed flow rates are quite small relative to working volume. Ensuring approximately equal volume flow rates through multiple feed points may be complex, but a thorough coverage of the working volume with the feed points would probably be less sensitive to differences in flow rates.

## 6 CONCLUSIONS

Optimal placement of multiple feed points was derived from 1D diffusion equations. The effect on both mixing and bioreaction in industrially relevant stirred tank and bubble column bioreactors was then evaluated with compartment model simulations. Placing multiple feed points at the middle height of equal‐height axial subdivisions improved mixing and reaction substantially: Simulated mixing times were reduced from the scale of minutes to the scale of 10 s, which mitigated substrate gradients and restored ideal homogeneous reactor performance. The heterogeneity in pH and concentration of dissolved oxygen was reduced as well. The implications regarding bioreactor scale‐up are considerable. The use of appropriately placed feed points could homogenize large‐scale reactors effectively and consequently (1) alleviate one of the most cited scale‐up problems, the heterogeneity of substrate, oxygen, and pH, (2) reduce side‐product formation, (3) maintain optimal biomass and product yields, and (4) improve reactor control. Another benefit is that even the simple homogeneous ideal reactor model remains applicable when the large scale resembles the small.

## CONFLICT OF INTEREST

The authors declare no conflict of interest.

## Supporting information

Supplementary Information

## Data Availability

The data that support the findings of this study are available from the corresponding author upon reasonable request.

## APPENDIX A COMPARTMENT MODELING

The stirred reactors R1 and R4 with one and four Rushton turbines, respectively, were modeled by combining axisymmetric two‐dimensional (2D) compartment modeling approaches (Cui et al., 1996; Vrábel et al., 1999, 2000) with 2D/3D networks‐of‐zones models (Delafosse et al., 2014; Hristov et al., 2001; Zahradník et al., 2001). The radial flow pattern of each impeller was composed of two stacked square networks of nested flow loops (Delafosse et al., 2014; Hristov et al., 2001; Zahradník et al., 2001). Furthermore, one square network of nested loops was used at the top of the reactor to model the stagnant surface. The stagnant top was assumed to reach a quarter from the surface toward the top impeller, which is similar to previous works (Vrábel et al., 1999, 2000) and matches experimental observations (Cronin et al., 1994; Jaworski et al., 1996) as well. In the multi‐impeller reactor R4, the square networks reached from the impeller plane midway toward the next impeller. The compartments within a square network of nested loops were of equal volume (Cui et al., 1996; Hristov et al., 2001; Vrábel et al., 1999, 2000; Zahradník et al., 2001). The effect of turbulence was modeled by connecting adjacent rows of compartments by axial exchange flows of equal magnitude (Cui et al., 1996; Vrábel et al., 1999, 2000). The tangential dimension was incorporated by multiplying the described 2D pattern tangentially. A tangential circulation flow then connected the 2D axial‐radial patterns unidirectionally and formed closed loops around the reactor to model the swirling induced by the impellers (Delafosse et al., 2014; Hristov et al., 2001). Liquid volumes in the aerated R4's compartments were reduced by the global gas‐holdup.

Both R1's and R4's impeller‐wise total circulation flows were estimated to be $Q_{\;\text{C}\;}=1.5nD^{3}$ ($m^{3}\;s^{-1}$), similarly to Cui et al. (1996); Vrábel et al. (1999, 2000), where n is the stirrer speed ($s^{-1}$) and D the impeller diameter (m). The 80% averaging factor used by Cui et al. (1996) and Vrábel et al. (1999, 2000) was not used here, as the model's structure incorporated also the shorter flow paths compensated for by the averaging in the cited studies. Each impeller's total circulation flow rate was then divided equally between the nested flow loops (Delafosse et al., 2014; Hristov et al., 2001; Zahradník et al., 2001). The total tangential circulation flow in an impeller region was assumed to be equal to the impeller's radial flow pattern's circulation flow (Delafosse et al., 2014; Hristov et al., 2001), and it was also divided equally between the nested tangential loops. It has been suggested that circulation in the stagnant surface should be slower than in the top impeller region (Vrábel et al., 1999, 2000). Therefore, the stagnant top loop's circulation flow rate was assumed to be one eight of the top impeller's circulation flow, which resulted in a doubled circulation time relative to the top impeller. The exchange flows $Q_{\;\text{E}}=N_{\text{E}\;}nD^{3}$ ($m^{3}\;s^{-1}$) were kept equal throughout the reactors, and the exchange flow numbers were fitted to $N_{\;\text{E}\;}=0.539$ in R4 and $N_{\;\text{E}\;}=0.274$ in R1 by matching the model outputs to yield the reported locally measured mixing times of 165 s in R4 (Vrábel et al., 1999, 2001; Xu et al., 1999) and 124 s in R1 (Langheinrich et al., 1998). The fitted exchange flows were divided equally across the compartments on each of the reactors' cross‐section planes. It should be noted that the exchange flow numbers depend on the used compartment model structure (Alves et al., 1997; Cui et al., 1996; Vrábel et al., 1999).

Given that the gas‐induced flow rates are similar to the exhange flows in a stirred tank (Vrábel et al., 1999, 2000), they were not modeled separately here. In effect, the exchange flow number covered both exchange and gas‐induced flows. Such an approach is appropriate when the impellers are not flooded (Vrábel et al., 2000) and the gas flow is not heterogeneous, as is the case here: $u_{\;\text{G}\;}=0.923\;{\text{cm}}^{3}\;s^{-1}$ in R4 is well below the 3 ${\text{cm}}^{3}\;s^{-1}$ transition threshold suggested for general use by Nauha et al. (2015). Under flooding conditions, the compartment model structure would require a change toward a bubble column compartment structure (described below) for the lowest, flooded impeller (Alves & Vasconcelos, 1995; Machon & Jahoda, 2000; Nauha et al., 2018).

The bubble columns B6 and B13 were modeled similarly to the stirred tanks, but with single rectangular networks of nested flow loops (Zahradník et al., 2001). The bubble columns were conceptually divided into bottom, middle, and top parts (Figure 1). The bottom and top parts had heights equal to the tank diameter (Degaleesan et al., 1997), and the rest of the column was considered the middle part. The compartment volumes were kept equal within the bottom, middle, and top parts of the reactor. The overall circulation flow rates $Q_{\;\text{C}\;}$ were estimated by using a correlation for the average liquid upflow velocities (Degaleesan et al., 1997)

(A1)
$$\frac{u_{\;\text{L}\;}}{\text{cm}\;s^{-1}}=2.2{\left(\frac{u_{\;\text{G}\;}}{\text{cm}\;s^{-1}}\frac{T}{\text{cm}}\right)}^{0.4}.$$

The velocity was multiplied by half the reactor's cross‐section as, in general, approximately one half of the cross‐section flows up and the other half down (Degaleesan et al., 1997). In Equation (A1), $u_{\;\text{G}\;}$ is the superficial gas velocity and T the reactor's diameter. The circulation flow rates were then divided across the nested loops with a quadratic profile matching an experimentally determined radial profile of liquid velocity in the reactor's middle part (Degaleesan et al., 1997). Exchange flow rates $Q_{\;\text{E}\;}$ were determined by finite‐volume discretization (Versteeg & Malalasekera, 2007) of eddy diffusivities estimated from literature data. The gas‐holdups and eddy diffusivities were radially homogeneous in the uppermost row of the top part and downmost row of the bottom part, but both of these properties were smoothed in the axial direction linearly toward the radially heterogeneous profiles of the middle part. For comparison, Degaleesan et al. (1997) modeled the top and bottom parts as single homogeneous compartments. Axial mean eddy diffusivity $d_{z}$ was approximated with (Degaleesan et al., 1997)

(A2)
$$\frac{d_{z}}{{\text{cm}}^{2}\;s^{-1}}=106.6{\left(\frac{u_{\;\text{G}\;}}{\text{cm}\;s^{-1}}\frac{T}{\text{cm}}\right)}^{0.3}-2325{\left(\frac{\text{cm}}{T}\right)}^{0.8},$$

and radial with (Degaleesan et al., 1997)

(A3)
$$\frac{d_{r}}{{\text{cm}}^{2}\;s^{-1}}=13.0{\left(\frac{u_{\;\text{G}\;}}{\text{cm}\;s^{-1}}\frac{T}{\text{cm}}\right)}^{0.3}-350{\left(\frac{\text{cm}}{T}\right)}^{0.8}.$$

The radial profiles of axial and radial eddy diffusivities were modeled with linear and quadratic polynomial forms, respectively, approximating the measured distributions reported by Degaleesan et al. (1997). As tangential eddy diffusivity was not measured by Degaleesan et al. (1997), it was taken to be a quarter of the axial mean value, which is close to measurements reported by Dudukovic (2000). The tangential eddy diffusivity's radial profile was assumed to be the same as the radial eddy diffusivity's. The B6's overall gas‐holdup ($\epsilon_{\;\text{G}\;}=17\%$) was reported by Zahradník et al. (2001) and the B13's mean gas‐holdup ($\epsilon_{\;\text{G}\;}=23\%$) was estimated with the correlation (Degaleesan et al., 1997)

(A4)
$$\epsilon_{\;\text{G}\;}=0.07{\left(\frac{u_{\;\text{G}\;}}{\text{cm}\;s^{-1}}\right)}^{0.474-0.00626\frac{T}{\text{cm}}}.$$

The radial profiles of gas‐holdup were modeled according to the quadratic profile observed experimentally by Degaleesan et al. (1997). Axially the gas‐holdups were corrected by hydrostatic pressure (water density 997 $\text{kg}\;m^{-3}$) such that the reactor's overall gas‐holdups were not changed. Ideal gas behavior and 101,325 Pa head‐space pressure were assumed in the corrections. Liquid volumes in the compartments were reduced by the local gas‐holdups.

Five nested loops (10 columns radially) per network and 12 tangential coordinates were used for each reactor. Both stirred tanks were modeled with two stacked square networks (altogether 20 compartments axially) per impeller and a single square network for the stagnant surface above the top impeller. The bubble columns were modeled with single rectangular networks with 62 and 30 rows in the B13 and B6 reactors, respectively. In total, the reactors were modeled with 3600–10,800 compartments.

In terms of computation requirements, the compartment models yield much smaller systems (3600–10,800 computational cells) to be solved than CFD (100,000 cells, even 1,000,000 cells). Compared to a CFD‐based approach (Delafosse et al., 2014) where the compartment models are derived from a CFD‐determined flow field, the simple hydrodynamics approach (Cui et al., 1996; Degaleesan et al., 1997; Vrábel et al., 1999, 2000) enabled a flexible and quick programmatic construction of the compartment models. It should be noted that axial impellers could be modeled by the hydrodynamics approach as well (Vrábel et al., 2000).

## APPENDIX B pH‐CONTROL AND BIOREACTION SIMULATION RESULTS

Table B1

**Table B1:** Simulation results in an ideal homogeneous reactor and in reactors R4, R1, B13, and B6 with top feed and selected optimal multipoint feeds in the form "mean ± standard deviation.”

	Top/ideal	A1R1T1	A2R2T2	A4R2T2
pH	6
R4	5.55 ± 0.68	5.92 ± 0.27	6.00 ± 0.02	6.00 ± 0.00
R1	5.18 ± 0.64	5.25 ± 0.66	5.90 ± 0.32	5.94 ± 0.25
B13	5.44 ± 0.71	5.86 ± 0.40	6.00 ± 0.05	6.00 ± 0.04
B6	5.12 ± 0.62	5.42 ± 0.66	5.96 ± 0.18	5.98 ± 0.12
S/$\text{mg}\;L^{-1}$	16.7			
R4	34.0 ± 41.6	20.3 ± 16.8	17.1 ± 4.8	16.8 ± 2.3
R1	48.9 ± 121.0	38.3 ± 104.8	20.9 ± 21.0	19.6 ± 15.5
B13	33.4 ± 57.1	22.0 ± 28.8	17.6 ± 7.5	17.0 ± 4.4
B6	46.3 ± 116.9	35.7 ± 80.1	19.4 ± 16.9	18.5 ± 11.9
$\tau_{S}$/s	15
R4	21.2 ± 15.0	16.3 ± 6.0	15.2 ± 1.7	15.0 ± 0.8
R1	26.6 ± 43.5	22.8 ± 37.7	16.5 ± 7.5	16.0 ± 5.6
B13	21.0 ± 20.5	16.9 ± 10.4	15.3 ± 2.7	15.1 ± 1.6
B6	25.7 ± 42.1	21.8 ± 28.8	16.0 ± 6.1	15.7 ± 4.3
O/$\text{mg}\;L^{-1}$	0.113			
R4	2.18 ± 2.60	0.233 ± 0.186	0.120 ± 0.028	0.114 ± 0.009
$Y_{OS}^{-1}r_{O}$/$g\;L^{-1}\;h^{-1}$	2.12			
R4	1.77 ± 0.79	2.10 ± 0.14	2.12 ± 0.06	2.12 ± 0.07
$y_{XS}$/%	43.8			
R4	33.2 ± 18.5	42.8 ± 2.7	43.6 ± 0.8	43.7 ± 0.4
R1	32.7 ± 22.2	38.4 ± 11.4	42.7 ± 3.5	42.9 ± 2.9
B13	37.2 ± 12.1	42.7 ± 3.1	43.5 ± 1.2	43.7 ± 0.7
B6	34.3 ± 19.9	35.4 ± 19.3	43.3 ± 1.6	43.4 ± 1.5

*Note*: Symbols: O, dissolved oxygen concentration; $Y_{OS}^{-1}r_{O}$, aerobic substrate consumption rate; S, substrate concentration; $\tau_{S}$, substrate consumption time‐scale; $y_{XS}$, biomass yield.
