# Supplementary material for: Substantial gradient mitigation in simulated large‐scale bioreactors by optimally placed multiple feed points
Source: Biotechnol Bioeng. 2022 Sep 26;119(12):3549–66. doi: 10.1002/bit.28232 (PMC9828524; doi:10.1002/bit.28232)
Supplement: Supplementary file 1 — Supplementary Information [file BIT-119-3549-s001.pdf]

Supporting information for Substantial gradient mitigation in simulated large-scale bioreactors by optimally placed multiple feed points

Pauli Losoi [*] Jukka Konttinen Ville Santala

Faculty of Engineering and Natural Sciences, Tampere University, Hervanta campus, Korkeakoulunkatu 8, Tampere, 33720, Finland

[*] Corresponding author, pauli.losoi@tuni.fi

Table S1: Bioreaction simulation results in an ideal homogeneous reactor and in reactors R4, R1, B13, and B6 with top feed and selected optimal multi-point feeds in the form "mean ± standard deviation" with $X = 50\,\mathrm{g\,L^{-1}}$ biomass concentration. Other kinetic parameters are exactly the same as in Table 2 of main text.

	Top/ideal	A1R1T1	A2R2T2	A4R2T2
S / $\mathrm{mg\,L^{-1}}$	2.17			
R4	4.14 ± 15.65	3.28 ± 10.66	2.44 ± 3.36	2.28 ± 1.99
R1	13.1 ± 94.8	12.5 ± 92.9	4.18 ± 15.48	3.51 ± 10.39
B13	7.19 ± 40.90	4.65 ± 22.37	2.71 ± 5.39	2.42 ± 3.29
B6	13.9 ± 97.1	9.22 ± 64.97	3.72 ± 12.85	3.12 ± 8.86
τ_S / s	1.82			
R4	2.10 ± 1.13	2.04 ± 0.77	1.98 ± 0.24	1.96 ± 0.14
R1	2.74 ± 6.82	2.70 ± 6.69	2.10 ± 1.11	2.05 ± 0.75
B13	2.32 ± 2.94	2.13 ± 1.61	2.00 ± 0.39	1.97 ± 0.24
B6	2.80 ± 6.99	2.46 ± 4.68	2.07 ± 0.93	2.02 ± 0.60
O / $\mathrm{mg\,L^{-1}}$	0.113			
R4	7.00 ± 4.70	3.35 ± 2.68	0.658 ± 0.544	0.180 ± 0.101
$Y_{OS}^{-1} r_O$ / $\mathrm{g\,L^{-1}\,h^{-1}}$	2.12			
R4	0.961 ± 1.461	1.58 ± 1.67	2.03 ± 0.98	2.11 ± 0.54
y_{XS} / %	18.7			
R4	−357 000 ± 797 000	−473 ± 754	−14.9 ± 50.0	11.6 ± 15.3
R1	−1 770 000 ± 8 080 000	−779 000 ± 304 000	−526 ± 1380	−405 ± 1250
B13	−345 000 ± 919 000	−1600 ± 4290	−66.1 ± 126.3	0.163 ± 29.684
B6	−426 000 ± 1 897 000	−164 000 ± 814 000	−191 ± 395	−51.0 ± 98.2

Symbols: O, dissolved oxygen concentration; $Y_{OS}^{-1} r_O$, aerobic substrate consumption rate; S, substrate concentration; τ_S, substrate consumption time-scale; y_{XS}, biomass yield.

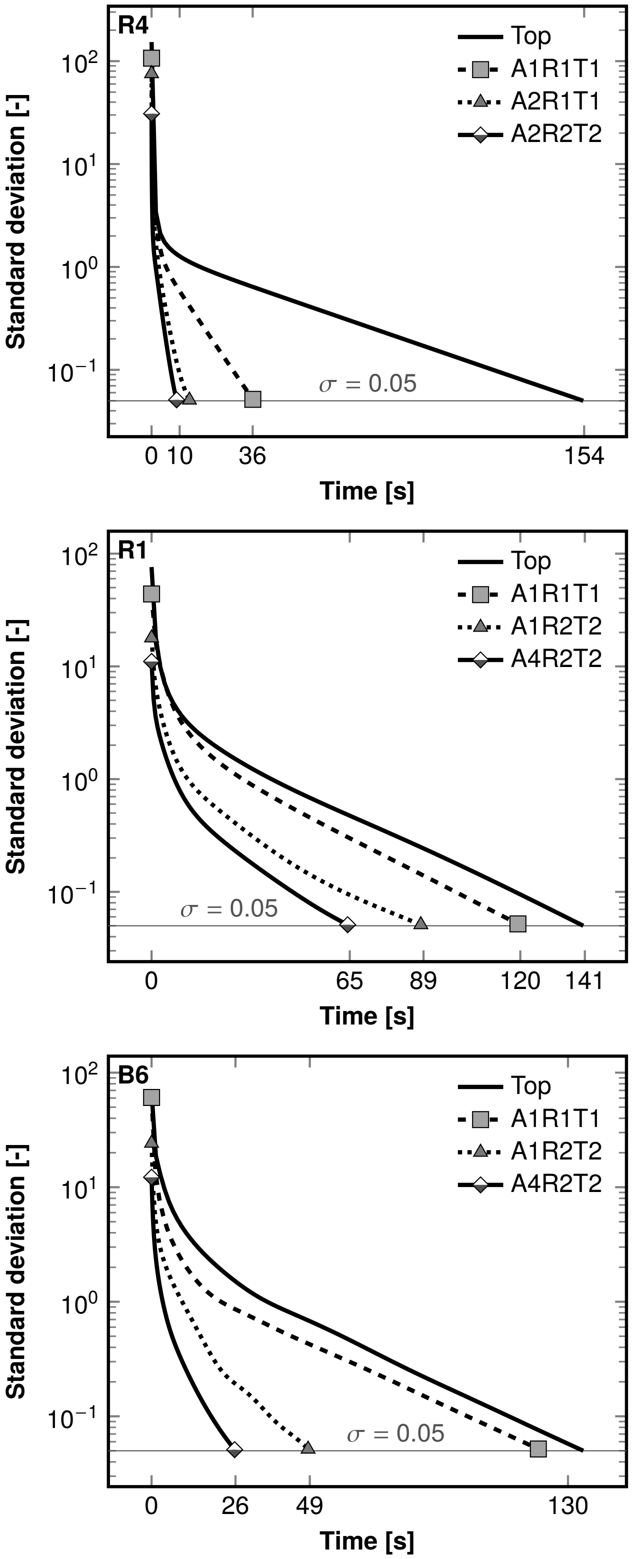

Figure S1: Standard deviation of dimensionless tracer concentration in reactors R4 (top), R1 (middle), and B6 (bottom) after tracer pulse with top feed and various optimal multi-point feeds. The feed arrangements AxRyTz contain x axial, y radial, and z tangential coordinates (see Figure 3 in main text). The 5 % line is the threshold for mixing time. Note the logarithmic scaling of the vertical axis.

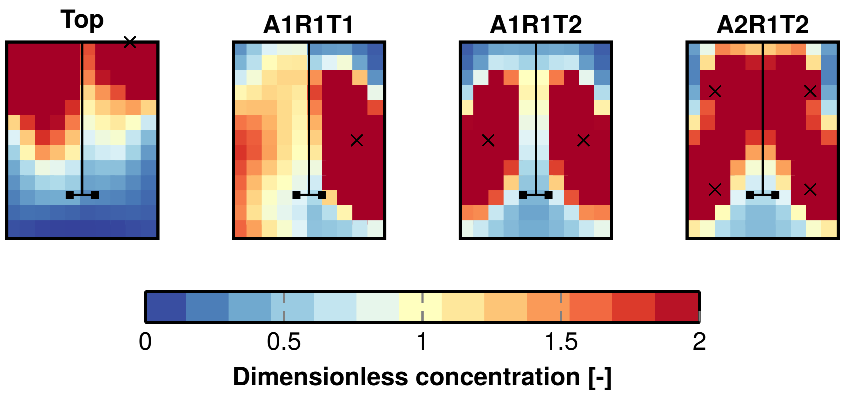

Figure S2: Simulated concentration of substrate in reactor R1 with $10\,\mathrm{g\,L^{-1}}$ biomass concentration and a $4\,\mathrm{g\,L^{-1}\,h^{-1}}$ substrate feed rate at the top or through an optimal multi-point feed. The concentrations have been normalized by the respective substrate concentration $16.7\,\mathrm{mg\,L^{-1}}$ calculated in an ideal homogeneous reactor at the same conditions. The feed arrangements AxRyTz contain x axial, y radial, and z tangential coordinates (see Figure 3 in main text).

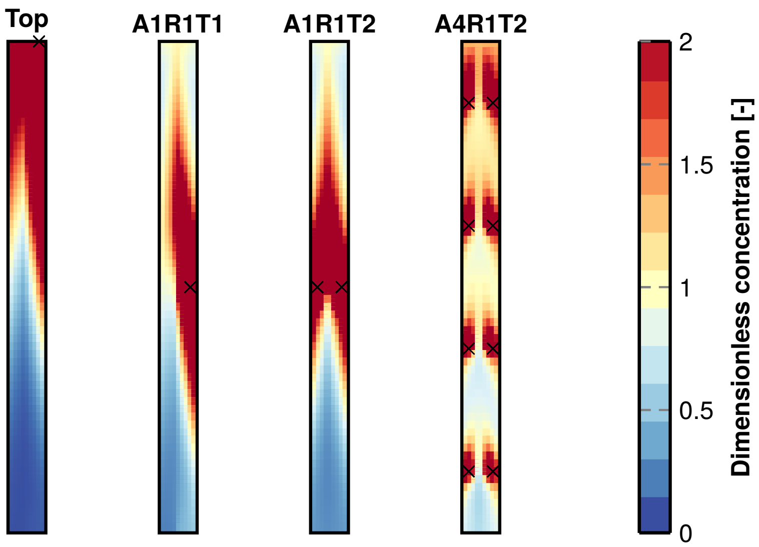

Figure S3: Simulated concentration of substrate in reactor B13 with $10\,\mathrm{g\,L^{-1}}$ biomass concentration and a $4\,\mathrm{g\,L^{-1}\,h^{-1}}$ substrate feed rate at the top or through an optimal multi-point feed. The concentrations have been normalized by the respective substrate concentration $16.7\,\mathrm{mg\,L^{-1}}$ calculated in an ideal homogeneous reactor at the same conditions. The feed arrangements AxRyTz contain x axial, y radial, and z tangential coordinates (see Figure 3 in main text).

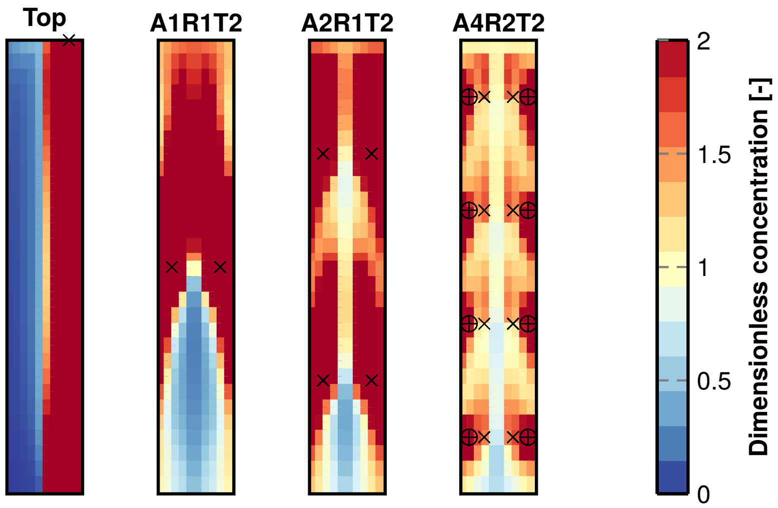

Figure S4: Simulated concentration of substrate in reactor B6 with $10\,g\,L^{-1}$ biomass concentration and a $4\,g\,L^{-1}\,h^{-1}$ substrate feed rate at the top or through an optimal multi-point feed. The concentrations have been normalized by the respective substrate concentration $16.7\,mg\,L^{-1}$ calculated in an ideal homogeneous reactor at the same conditions. The feed arrangements AxRyTz contain x axial, y radial, and z tangential coordinates (see Figure 3 in main text).

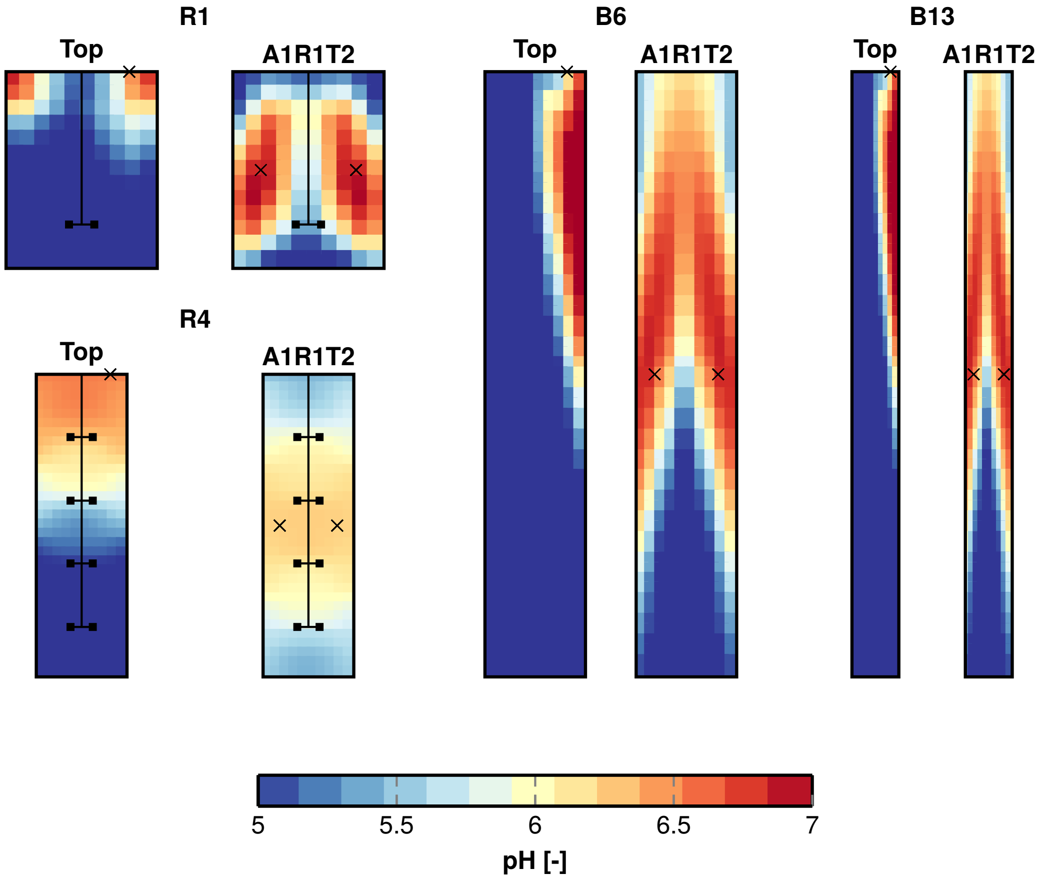

Figure S5: Simulated pH in reactors R1 (upper left), R4 (lower left), B6 (center), and B13 (right) 10 s after pH-correcting pulse of carbonate in a $100\,mmol\,L^{-1}$ carbonate buffer solution initially at a pH of 4.8. Responses to top and A1R1T2 pulse are shown. The A1R1T2 feed arrangement contains two feed points tangentially opposite to each other at middle height and 63 % radius (see Figure 3 in main text).

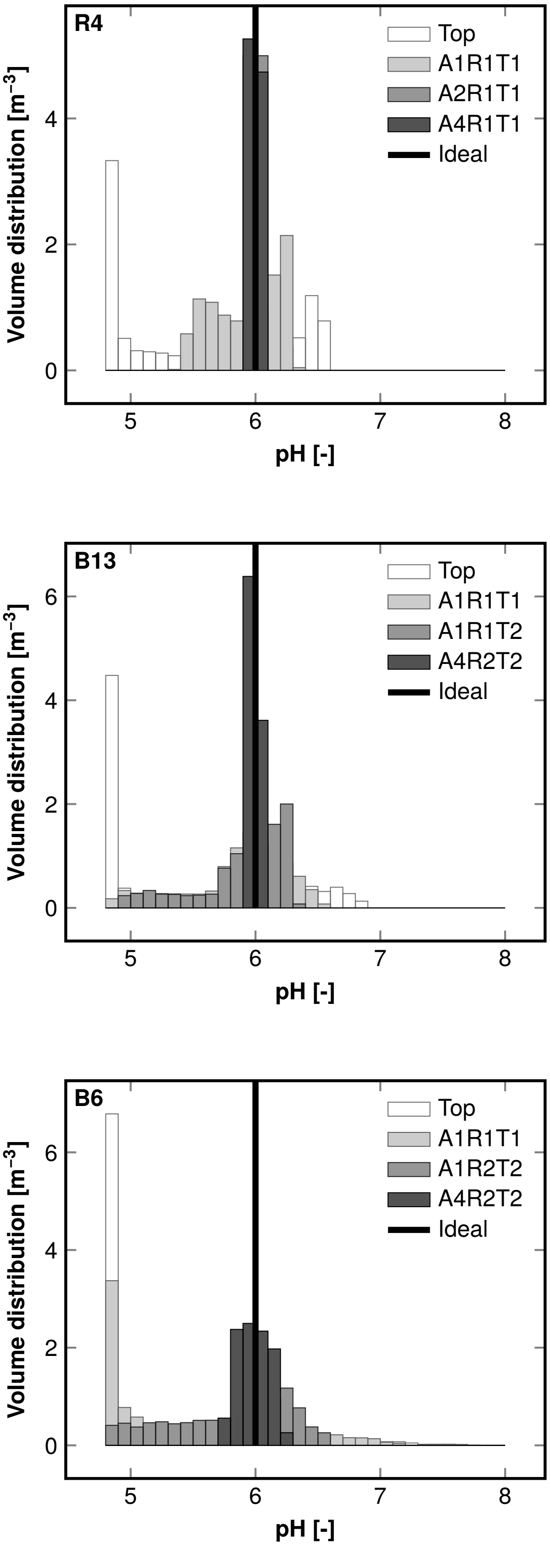

Figure S6: Distribution of pH in reactors R4 (top), B13 (middle), and B6 (bottom) 10 s after pH-correcting pulse of carbonate in a 100 mmol L^{-1} carbonate buffer solution initially at a pH of 4.8. Responses to top and optimal multi-point pulses are shown. Ideal homogeneous reactor behaviour is shown for reference (whole volume has pH 6). The feed arrangements AxRyTz contain x axial, y radial, and z tangential coordinates (see Figure 3 in main text).

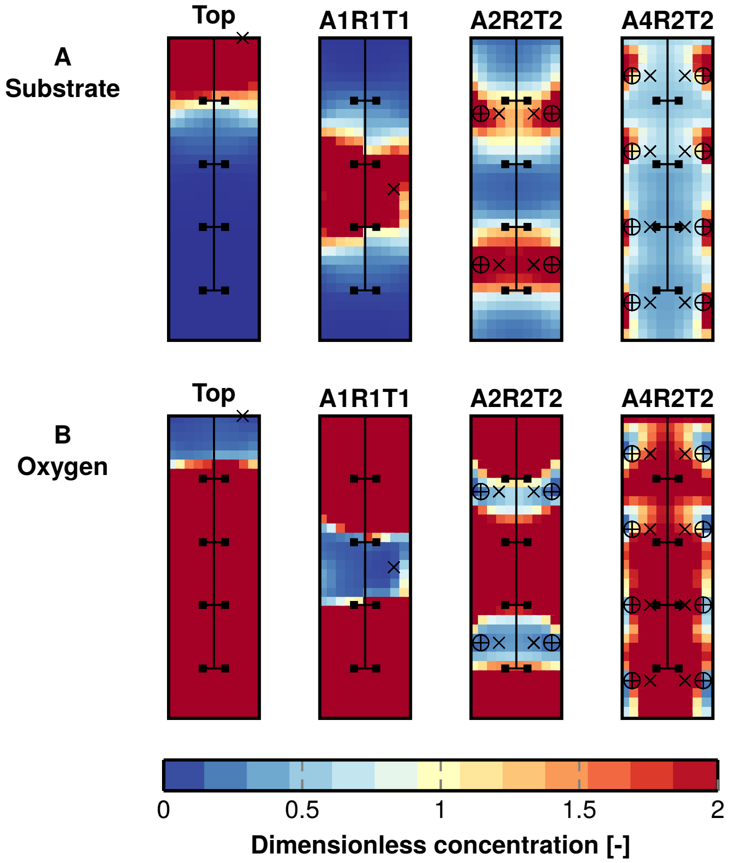

Figure S7: Simulated concentration of substrate and dissolved oxygen in reactor R4 with $50\,\mathrm{g\,L^{-1}}$ biomass concentration and a $4\,\mathrm{g\,L^{-1}\,h^{-1}}$ substrate feed rate at the top or through an optimal multi-point feed. The concentrations have been normalized by respective concentrations (substrate $2.17\,\mathrm{mg\,L^{-1}}$, oxygen $0.113\,\mathrm{mg\,L^{-1}}$) calculated in an ideal homogeneous reactor at the same conditions. Note the color scale limits (values above twice the ideal reactor value are shown as 2). The feed arrangements AxRyTz contain x axial, y radial, and z tangential coordinates (see Figure 3 in main text). The substrate gradients were similar in the other reactors (R1, B13, and B6) as well (not shown).

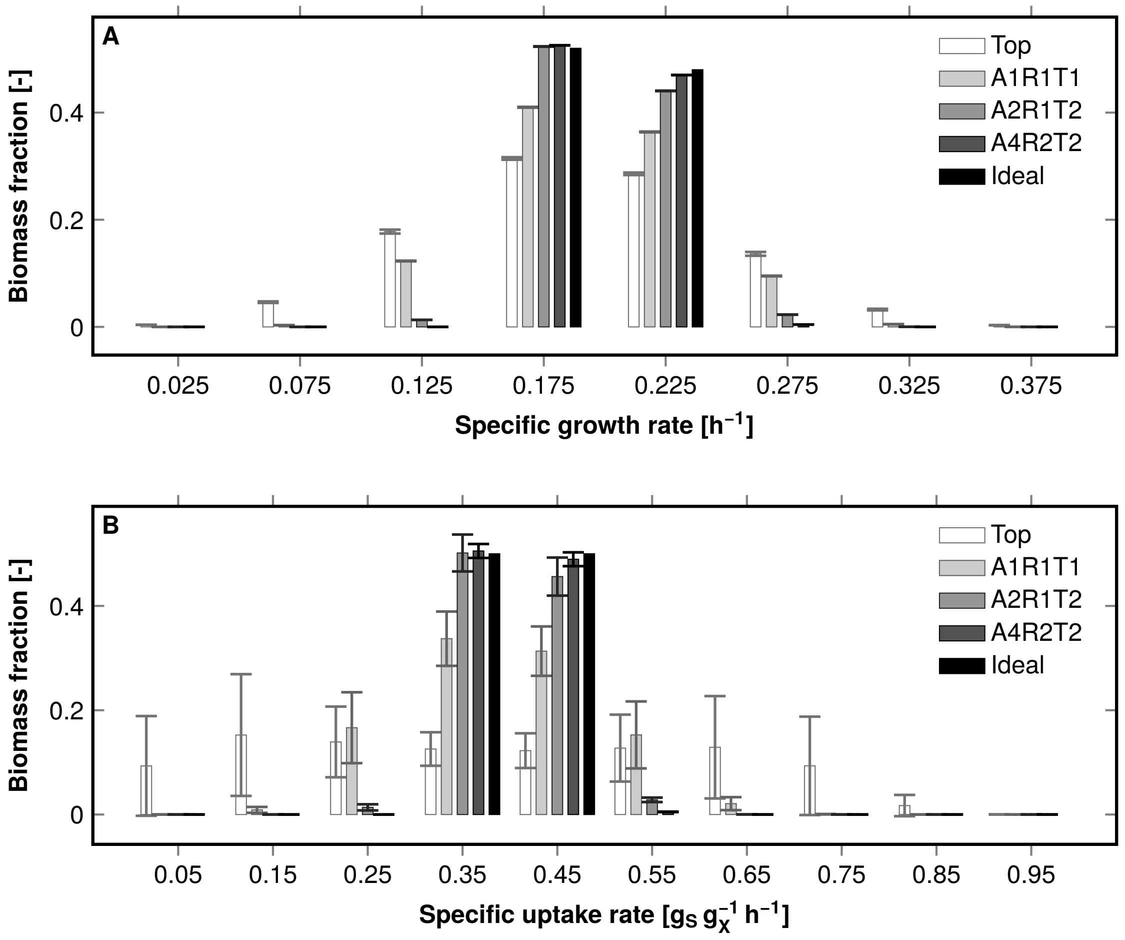

Figure S8: Simulated distribution of biomass-specific growth (A) and substrate uptake (B) rates in reactor R4 with $10\,g\,L^{-1}$ biomass concentration and a $4\,g\,L^{-1}\,h^{-1}$ substrate feed at the top or through an optimal multi-point feed. In panel A the two highest classes with only negligible biomass are not shown. The error bars represent volumetric standard deviations. The feed arrangements AxRyTz contain x axial, y radial, and z tangential coordinates (see Figure 3 in main text). The ideal homogeneous reactor results are shown for reference.

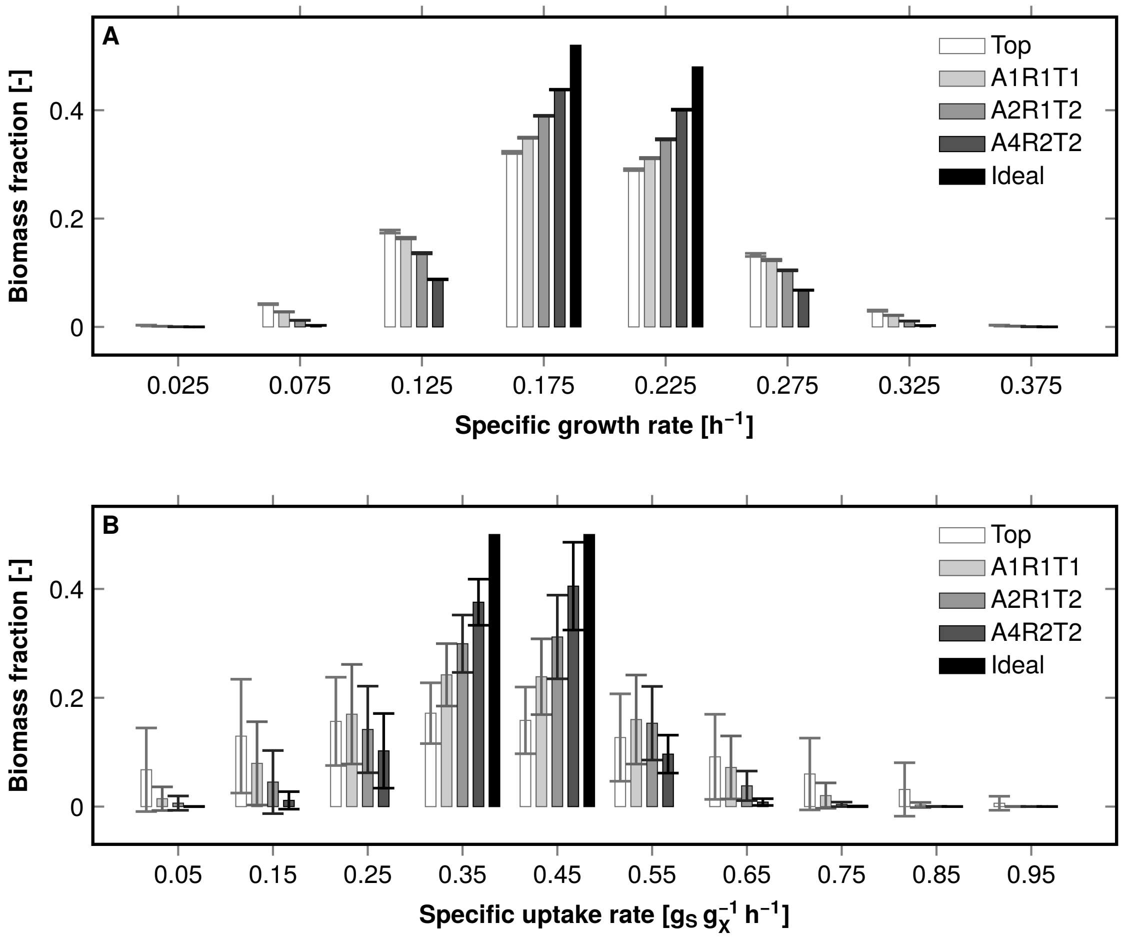

Figure S9: Simulated distribution of biomass-specific growth (A) and substrate uptake (B) rates in reactor R1 with $10\,g\,L^{-1}$ biomass concentration and a $4\,g\,L^{-1}\,h^{-1}$ substrate feed at the top or through an optimal multi-point feed. In panel A the two highest classes with only negligible biomass are not shown. The error bars represent volumetric standard deviations. The feed arrangements AxRyTz contain x axial, y radial, and z tangential coordinates (see Figure 3 in main text). The ideal homogeneous reactor results are shown for reference.

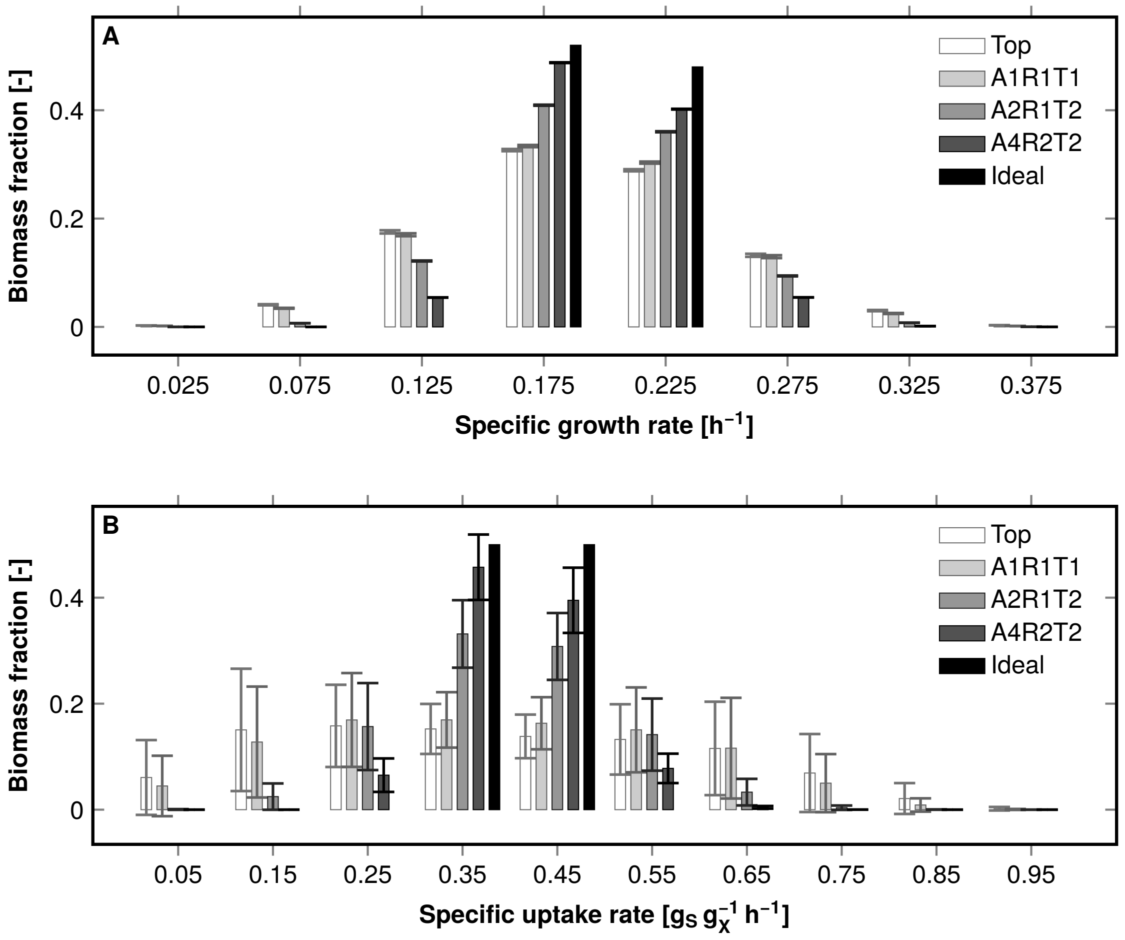

Figure S10: Simulated distribution of biomass-specific growth (A) and substrate uptake (B) rates in reactor B6 with $10\,g\,L^{-1}$ biomass concentration and a $4\,g\,L^{-1}\,h^{-1}$ substrate feed at the top or through an optimal multi-point feed. In panel A the two highest classes with only negligible biomass are not shown. The error bars represent volumetric standard deviations. The feed arrangements AxRyTz contain x axial, y radial, and z tangential coordinates (see Figure 3 in main text). The ideal homogeneous reactor results are shown for reference.

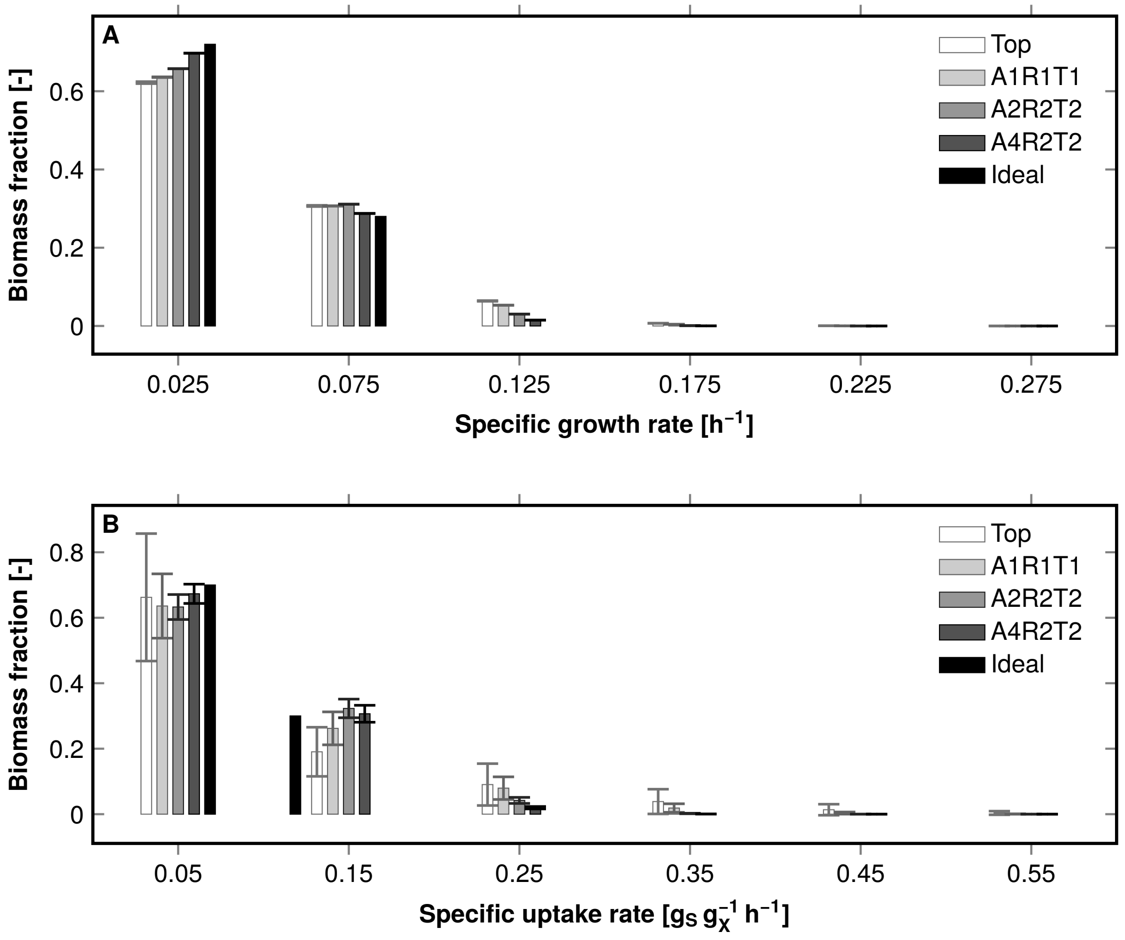

Figure S11: Simulated distribution of biomass-specific growth (A) and substrate uptake (B) rates in reactor B13 with 50 g L^{-1} biomass concentration and a 4 g L^{-1} h^{-1} substrate feed at the top or through an optimal multi-point feed. The four highest classes with only negligible biomass are not shown. The error bars represent volumetric standard deviations. The feed arrangements AxRyTz contain x axial, y radial, and z tangential coordinates (see Figure 3 in main text). The ideal homogeneous reactor results are shown for reference. The results were similar in the other reactors (R4, R1, and B6) as well (not shown).
